# Intrinsic Cellular Properties and Connectivity Density Determine Variable Clustering Patterns in Randomly Connected Inhibitory Neural Networks

**DOI:** 10.3389/fncir.2016.00082

**Published:** 2016-10-20

**Authors:** Scott Rich, Victoria Booth, Michal Zochowski

**Affiliations:** ^1^Applied and Interdisciplinary Mathematics, University of MichiganAnn Arbor, MI, USA; ^2^Departments of Mathematics and Anesthesiology, University of MichiganAnn Arbor, MI, USA; ^3^Departments of Physics and Biophysics, University of MichiganAnn Arbor, MI, USA

**Keywords:** inhibitory networks, interneurons, phase response curve, computational model, clustering, synchrony, M-current, spike-frequency adaptation

## Abstract

The plethora of inhibitory interneurons in the hippocampus and cortex play a pivotal role in generating rhythmic activity by clustering and synchronizing cell firing. Results of our simulations demonstrate that both the intrinsic cellular properties of neurons and the degree of network connectivity affect the characteristics of clustered dynamics exhibited in randomly connected, heterogeneous inhibitory networks. We quantify intrinsic cellular properties by the neuron's current-frequency relation (IF curve) and Phase Response Curve (PRC), a measure of how perturbations given at various phases of a neurons firing cycle affect subsequent spike timing. We analyze network bursting properties of networks of neurons with Type I or Type II properties in both excitability and PRC profile; Type I PRCs strictly show phase advances and IF curves that exhibit frequencies arbitrarily close to zero at firing threshold while Type II PRCs display both phase advances and delays and IF curves that have a non-zero frequency at threshold. Type II neurons whose properties arise with or without an M-type adaptation current are considered. We analyze network dynamics under different levels of cellular heterogeneity and as intrinsic cellular firing frequency and the time scale of decay of synaptic inhibition are varied. Many of the dynamics exhibited by these networks diverge from the predictions of the interneuron network gamma (ING) mechanism, as well as from results in all-to-all connected networks. Our results show that randomly connected networks of Type I neurons synchronize into a single cluster of active neurons while networks of Type II neurons organize into two mutually exclusive clusters segregated by the cells' intrinsic firing frequencies. Networks of Type II neurons containing the adaptation current behave similarly to networks of either Type I or Type II neurons depending on network parameters; however, the adaptation current creates differences in the cluster dynamics compared to those in networks of Type I or Type II neurons. To understand these results, we compute neuronal PRCs calculated with a perturbation matching the profile of the synaptic current in our networks. Differences in profiles of these PRCs across the different neuron types reveal mechanisms underlying the divergent network dynamics.

## 1. Introduction

Inhibitory interneurons play a crucial role in the formation of rhythmic electrical activity throughout the brain. In the hippocampus, interneurons mediate rhythms that appear to serve vital roles in memory processing and are affected by sleep-wake activity (Traub et al., 1998; Kopell et al., 2000; Bartos et al., 2007; Aton et al., 2013). Interneurons also play a key role in generating rhythmic activity in the cortex; in the visual cortex in particular, these rhythms are implicated in the control of attention in the presence of competing stimuli (Desimone and Duncan, 1995; Luck et al., 1997; Reynolds et al., 1999; Fries, 2005; Bosman et al., 2012).

However, interneurons form a very heterogenous population. For example, the oriens-lacunosum moleculare (OLM) cells of the hippocampus contain an M-type potassium current which causes spike-frequency adaptation and is blocked by the action of acetylcholine (ACh) on muscarinic receptors (Saraga et al., 2003; Lawrence et al., 2006; Cutsuridis et al., 2010; Cutsuridis and Hasselmo, 2012). Other hippocampal interneurons, such as the parvalbumin-containing basket cells (PV cells) (Ferguson et al., 2013) and cholecystokinin-containing basket cells (CCK cells) (Cea-del Rio et al., 2011, 2012), have distinct cellular properties: the PV cells are fast spiking neurons without adaptation, while the CCK cells exhibit cholinergic modulation. In contrast, in the cortex, cells exhibiting the PV marker exhibit a wide range of properties, including the possibility of expressing the M-type potassium channel, while somatostatin-expressing interneurons (SOM cells) consistently exhibit spike frequency adaptation much like the OLM cells (Markram et al., 2004; Perrenoud et al., 2013).

Many of the properties of these interneurons can be encapsulated by the neuron's current-frequency relation (IF curve) and Phase Response Curve (PRC), the latter being a measure of how perturbations to a neuron at various phases of its periodic firing cycle affect the timing of the next action potential (Hansel et al., 1995; Schemer and Lewis, 2012). The neuron properties quantified by these two tools are very often related, leading to the development of the classical Type I and Type II neuron classifications: neurons classified as Type I exhibit a steep IF curve with an arbitrarily low firing frequency and a PRC (calculated with a brief, weak excitatory perturbation) exhibiting only phase advances; in contrast, neurons classified as Type II exhibit a more shallow IF curve with a minimum non-zero firing frequency and a PRC exhibiting regions of phase delay and advance (Wang, 2010). These classifications arose historically from the concepts of excitability type (Hodgkin, 1948) and the type of bifurcation that leads to periodic firing in the corresponding mathematical models. While recent work has shown that the relationship between the PRC, the IF curve and the bifurcation type are not definite (Ermentrout et al., 2012), usually saddle-node (SNIC) bifurcations are associated with Type I properties and subcritical Hopf bifurcations are associated with Type II properties (Ermentrout et al., 2001; Stiefel et al., 2008). Additionally, the presence of an M-type adaptation current has been shown to change Type I neurons into Type II neurons through a corresponding change in the bifurcation type (Ermentrout et al., 2001; Stiefel et al., 2008).

Computational studies of networks of biophysical neuron models have played a large role in identifying the mechanisms by which inhibitory interneurons generate rhythmic firing amongst themselves and in synaptically connected excitatory pyramidal cells. Previous studies on the rhythmic properties of strictly inhibitory networks have primarily focused on networks consisting of Type I neurons (Wang and Buzsáki, 1996; Chow et al., 1998; Whittington et al., 2000; Bartos et al., 2002; Brunel and Hansel, 2006). The PV cells of the hippocampus have been shown to exhibit a PRC only showing phase advance in response to a weak excitatory current pulse and thus are typically classified as Type I; these neurons are also known to exhibit reciprocal synapses to form an inhibitory network primarily containing only this type of interneuron (Karson et al., 2009; Ferguson et al., 2013). The tendency for these networks to synchronize has been explained by the interneuron network gamma (ING) mechanism (Whittington et al., 2000; Kopell et al., 2010; Wang, 2010). Synchrony is initiated by this mechanism when each neuron in the network receives synaptic inhibitory input at roughly the same time that suppresses firing and keeps each neuron's membrane potential below firing threshold. When the synaptic inhibition decays, this creates an optimal firing window in which neurons can fire action potentials. Thus, a major feature of this mechanism is that inhibitory signals promote synchronization by gating the timing of neural firing. As a result, synchrony via the ING mechanism is sensitive to properties of the synaptic currents present in the network and is most robust when networks are densely connected and cellular heterogeneity is low (Traub et al., 1998; Whittington et al., 2000; Tiesinga and Sejnowski, 2009; Kopell et al., 2010; Wang, 2010). In particular, the ING mechanism predicts that synaptic inhibition that is sufficiently strong and long lasting should robustly cause synchrony amongst intrinsically firing cells whose firing frequencies are similar.

Type II neurons have been analytically shown to synchronize in the case of mutual inhibition in networks of two neurons (Vreeswijk et al., 1994). Other studies have analyzed larger, all-to-all coupled inhibitory networks of neurons exhibiting these properties, showing that they can either exhibit synchrony or anti-phase clustering (Hansel et al., 1995; Achuthan and Canavier, 2009; Ladenbauer et al., 2012), while newer studies have begun to investigate the activity of these types of neurons in randomly connected networks (Viriyopase et al., 2016). Oftentimes, the presence of adaptation currents (like the M-type potassium current) is what imbues a neuron with Type II properties, as is the case in the neurons studied by Ladenbauer et al. Hippocampal OLM cells exhibit Type II properties while also exhibiting spike-frequency adaptation, imparted by the M-type potassium current (Saraga et al., 2003; Lawrence et al., 2006; Cutsuridis et al., 2010; Cutsuridis and Hasselmo, 2012), and the SOM cells and some interneurons expressing the PV marker exhibit these properties in the cortex (Markram et al., 2004; Perrenoud et al., 2013). However, neurons may feature Type II properties without containing adaptation currents, as is most simply illustrated by the classic Hodgkin-Huxley model neuron which does not exhibit spike-frequency adaptation but does exhibit Type II properties (Hodgkin and Huxley, 1952; Ermentrout and Terman, 2010). Interneurons exhibiting Type II properties without strong evidence of an adaptation current have been found in various brain regions including the rat somatosensory cortex (Tateno et al., 2004; Tateno and Robinson, 2007a,b), the rat barrel cortex (Mancilla et al., 2007), the rat cerebellum (Couto et al., 2015) and the mouse spinal cord (Zhong et al., 2012). There is evidence suggesting that Type II interneurons in the rat somatosensory cortex synapse onto each other to form an inhibitory network primarily containing only this type of interneruon (Tateno and Robinson, 2007b), and the PV cells in the cortex, which sometimes exhibit Type II properties, are also known to be connected in this fashion (Markram et al., 2004; Perrenoud et al., 2013); however, it is currently unclear whether the OLM interneurons are synaptically interconnected.

Motivated by the variety of interneurons present in the hippocampus and cortex, and evidence that they form inhibitory networks, in this study we investigated spatio-temporal pattern formation in strongly synaptically coupled, randomly connected inhibitory networks of Type I neurons, Type II neurons and Type II neurons containing an M-type potassium current (hereafter referred to as Type II neurons with adaptation). We focus on this coupling regime because networks of this type are not amenable to analytical treatment. Utilizing simulations, we show that these networks exhibit different types of synchronous or clustering behavior through multiple mechanisms, which arise from the differing intrinsic cellular properties of these neuron models.

**Figure 1:**
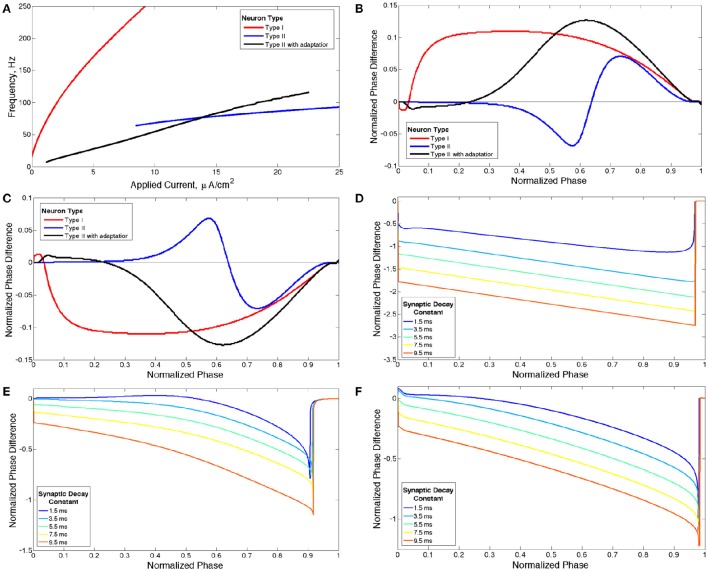
**Properties of neuron models**. **(A)** Current-frequency curves (IF curves) of Type I (red), Type II (blue) and Type II with adaptation (black) neuron models. **(B)** Phase response curves (PRCs) calculated with a brief excitatory current pulse for each model neuron firing at 65 Hz. **(C)** PRCs calculated with a brief inhibitory pulse for each model neuron neuron firing at 65 Hz. **(D–F)** sPRCs calculated with a perturbation matching the double exponential synaptic current model with various synaptic decay constants for a Type I neuron firing at 44 Hz **(D)**, for a Type II neuron firing at 70 Hz **(E)** and for a Type II neuron with adaptation firing at 30 Hz **(F)**.

Because the neuron models used in this study exhibit the classical associations between the PRC, IF curve and bifurcation type (shown below) (Ermentrout et al., 2001; Stiefel et al., 2008), we will refer to Type I and Type II neurons and PRCs following the classical definitions described above. While we use PRCs generated with a weak excitatory current pulse to classify our model neurons as either Type I or Type II, we use a PRC calculated with a perturbation matching the synaptic current profile, which we term the sPRC and define in more detail below, to more accurately illustrate neural response properties in our networks which contain stronger synaptic connections. Neurons that exhibit distinct properties in their PRCs exhibit analagously distinct properties in their sPRCs as illustrated in Figure 1, and these sPRC properties are used to articulate the mechanisms underlying the dynamics found in these networks.

We classify network activity patterns into four major behaviors: asynchrony; full synchrony, in which every neuron in the network fires roughly simultaneously in a stable fashion; one-cluster dynamics, in which some of the neurons in the network fire synchronously in bursts of network activity, but others are silenced; and two-cluster dynamics, in which some, but not all, of the neurons in the network fire synchronously in bursts, but subsequent bursts contain mutually exclusive populations of neurons, providing informational specificity to the burst. While a number of these dynamical patterns have previously been found in computational studies of neural networks (Talathi et al., 2008; Ermentrout and Wechselberger, 2009; Talathi et al., 2010; Kilpatrick and Ermentrout, 2011; Dipoppa et al., 2012; Moon et al., 2015), we focus on directly comparing network activity in large-scale, synaptically coupled inhibitory networks consisting of neurons with different membrane properties and with differing cellular heterogeneity and connectivity density.

Our results show that the ING mechanism drives one-cluster dynamics via cell suppression (Chow et al., 1998) and full synchrony in networks of Type I neurons, as has been previously shown (Whittington et al., 2000; Kopell et al., 2010; Wang, 2010). However, we also show that the properties of the sPRC of the neuron, specifically those associated with Type II neurons, can interfere with the ING mechanism and produce two-cluster dynamics. The fact that such networks do not necessarily evolve into one-cluster dynamics as the synaptic decay time constant increases violates the predictions of the ING mechanism and our results indicate that clustering in these networks occurs in a fashion largely independent of synaptic properties. In fact, ING-driven synchrony only appears in networks of Type II neurons when heterogeneity in the intrinsic firing frequency of cells in the network is minimal. Previous studies have shown that the PRC is a useful tool to explain divergent network dynamics, including the differences between one-cluster and two-cluster firing, although such analysis has not been performed in detail on strongly and randomly connected, strictly inhibitory networks, and has not utilized the features of the PRC focused on in this study (Ermentrout et al., 2001; Goel and Ermentrout, 2002; Talathi et al., 2008; Ermentrout and Wechselberger, 2009; Zahid and Skinner, 2009; Kilpatrick and Ermentrout, 2011; Ladenbauer et al., 2012; Canavier et al., 2013; Viriyopase et al., 2016).

Furthermore, we illustrate that networks of Type II neurons with adaptation exhibit activity patterns similar to either Type I or Type II networks dependent upon the average intrinsic cell firing frequency of neurons in the network and the synaptic decay constant of synapses in the network. The values of these parameters and their interaction with the properties of the adaptation current lead to a change in network dynamics that is associated with a change in properties of sPRC. These results show the importance of the adaptation current in driving network dynamics in strictly inhibitory, randomly-connected networks, further emphasizing the influence of spike-frequency adaptation on network dynamics as shown in other network types (Ermentrout et al., 2001; van Vreeswijk and Hansel, 2001; Ermentrout and Wechselberger, 2009; Ladenbauer et al., 2012; Viriyopase et al., 2016). Together, our results detail the important role played by intrinsic cellular properties of neurons, as well as the degree of connectivity in the network, in driving rhythmic behavior in randomly-connected inhibitory networks.

## 2. Methods

### 2.1. Neuron models

We constructed networks composed of three different model neurons in the Hodgkin-Huxley formalism that displayed different properties in their IF curves and PRCs. All model neurons contained Na^+^, K^+^-delayed rectifier and leak currents. The Type II neuron with adaptation additionally contained a slow, M-type K^+^ current (Stiefel et al., 2008; Ermentrout and Terman, 2010; Fink et al., 2011).

To model an interneuron exhibiting Type II properties without spike-frequency adaptation, the classic Hodgkin-Huxley equations were used (Hodgkin and Huxley, 1952; Ermentrout and Terman, 2010):

(1) $$\begin{array}{rcl} \frac{dV}{dt} & = & -g_{Na}m^{3}h\left(V-E_{Na}\right)-g_{K}n^{4}\left(V-E_{K}\right)-g_{L}\left(V-E_{L}\right)\\ \;+\;I_{app}-I_{syn} \end{array}$$

(2) $$\begin{array}{rcl} \frac{dX}{dt} & = & \alpha_{X}\left(V\right)\left(1-X\right)-\beta_{X}\left(V\right)X,\text{for}X=m,h,n \end{array}$$

(3) $$\begin{array}{rcl} \alpha_{m}\left(V\right) & = & -0.1\left(\frac{V+40}{e^{-\left(V+40\right)∕10}-1}\right) \end{array}$$

(4) $$\begin{array}{rcl} \beta_{m}\left(V\right) & = & 4e^{-\left(V+65\right)∕18} \end{array}$$

(5) $$\begin{array}{rcl} \alpha_{h}\left(V\right) & = & 0.07e^{-\left(V+65\right)∕20} \end{array}$$

(6) $$\begin{array}{rcl} \beta_{h}\left(V\right) & = & \frac{1.0}{e^{-\left(V+35\right)∕10}+1} \end{array}$$

(7) $$\begin{array}{rcl} \alpha_{n}\left(V\right) & = & -0.01\left(\frac{V+55}{e^{-\left(V+55\right)∕10}-1}\right) \end{array}$$

(8) $$\begin{array}{rcl} \beta_{n}\left(V\right) & = & 1.25e^{-\left(V+65\right)∕80} \end{array}$$

*V* represents the membrane voltage in [mV], while *m, n* and *h* represent the unitless gating variables of the ionic current conductances. *I*_*app*_ signifies the external applied current to the neuron (described below), in [μA/cm^2^], while *I*_*syn*_ describes the synaptic current input to the cell from the network (described below), also with units of [μA/cm^2^]. *E*_*Na*_, *E*_*K*_ and *E*_*L*_ are the reversal potentials, with *Na* symbolizing sodium, *K* symbolizing potassium, and *L* symbolizing the leak current. In this model these constants are set at *E*_*Na*_ = 50 mV, *E*_*K*_ = −77 mV and *E*_*L*_ = −54.4 mV. The corresponding maximum conductances *g*_*Na*_, *g*_*K*_ and *g*_*L*_ are set at *g*_*Na*_ = 120 mS/cm^2^, *g*_*K*_ = 36 mS/cm^2^ and *g*_*L*_ = 0.3 mS/cm^2^.

The Type II properties of this model neuron are reflected in its IF curve (Figure 1A) and PRC (Figure 1B) (Wang, 2010). At current threshold, firing frequency is a discrete, non-zero value and the slope of the IF curve is shallow for all applied current values. The discontinuity between a zero firing frequency and a non-zero firing frequency arises from the subcritical Hopf bifurcation that leads to periodic firing in this model neuron, and this bifurcation is also historically associated with the classification of this neuron model as Type II (Ermentrout et al., 2001; Stiefel et al., 2008). The PRC displays an initial delay region and phase advance when the brief depolarizing current pulse is delivered at later phases. The PRC calculated with a brief hyperpolarizing current pulse is simply a reflection over the line of zero phase difference, showing an initial phase advance region and phase delay when the perturbation is delivered at later phases (Figure 1C).

Type I neurons and Type II neurons with adaptation were simulated utilizing a model in which different values for the conductance associated with the M-type potassium current switch the behavior of the neuron between Type I and Type II with adaptation (Stiefel et al., 2008; Fink et al., 2011). The equations are

(9) $$\begin{array}{rcl} \frac{dV}{dt} & = & -g_{Na}m_{\infty }^{3}h\left(V-E_{Na}\right)-g_{K_{d}}n^{4}\left(V-E_{K}\right)\\ \;-\;g_{K_{s}}z\left(V-E_{K}\right)-g_{L}\left(V-E_{L}\right)+I_{app}-I_{syn} \end{array}$$

(10) $$\begin{array}{rcl} \frac{dX}{dt} & = & \frac{X_{\infty }\left(V\right)-X}{\tau_{X}\left(V\right)}\text{for}X=h,n,z \end{array}$$

(11) $$\begin{array}{rcl} m_{\infty }\left(V\right) & = & \frac{1}{1+e^{\left(-V-30∕9.5\right)}} \end{array}$$

(12) $$\begin{array}{rcl} h_{\infty }\left(V\right) & = & \frac{1}{1+e^{\left(V+53∕7.0\right)}} \end{array}$$

(13) $$\begin{array}{rcl} n_{\infty }\left(V\right) & = & \frac{1}{1+e^{\left(-V-30∕10\right)}} \end{array}$$

(14) $$\begin{array}{rcl} z_{\infty }\left(V\right) & = & \frac{1}{1+e^{\left(-V-39∕5\right)}} \end{array}$$

(15) $$\begin{array}{rcl} \tau_{h}\left(V\right) & = & 0.37+\frac{2.78}{1+e^{\left(V+40.5\right)∕6}} \end{array}$$

(16) $$\begin{array}{rcl} \tau_{n}\left(V\right) & = & 0.37+\frac{1.85}{1+e^{\left(V+27\right)∕15}} \end{array}$$

(17) $$\begin{array}{rcl} \tau_{z}\left(V\right) & = & 75 \end{array}$$

Variables and constants have identical meanings as in the Type II model, with the new terms *g*_*K*_*d*__ and *g*_*K*_*s*__ representing the maximal conductances associated with the delayed rectifier and slow M-type potassium currents, respectively, and *z* representing the gating variable governing the M-type potassium current. The constants for this model are as follows: *E*_*Na*_ = 55 mV, *E*_*K*_ = −90 mV, *E*_*L*_ = −60 mV, *g*_*Na*_ = 24 mS/cm^2^, *g*_*K*_*d*__ = 3 mS/cm^2^ and *g*_*L*_ = 0.02 mS/cm^2^.

When *g*_*K*_*s*__ = 0 mS/cm^2^ the model neuron is designated Type I because of the properties of its IF curve (Figure 1A) and PRC (Figure 1B) (Wang, 2010). The neuron exhibits firing frequencies arbitrarily close to zero at current threshold and the slope of the IF curve is initially very steep while becoming more shallow as applied current increases. The PRC exhibits phase advance for a brief depolarizing current pulse delivered at essentially every phase. When the PRC is calculated with a brief hyperpolarizing current pulse the curve is reflected over the line of zero phase difference, showing phase delay at essentially every phase (Figure 1C). This model neuron achieves repetitive firing via a SNIC bifurcation, which is historically associated with the Type I classification (Ermentrout et al., 2001; Stiefel et al., 2008).

When *g*_*K*_*s*__ = 1.5 mS/cm^2^ the model neuron is designated Type II with adaptation because the properties of its IF curve (Figure 1A) and PRC (Figure 1B) match the basic properties of a Type II neuron as described previously (Wang, 2010). This neuron model achieves periodic firing with a subcritical Hopf bifurcation, a known feature of neuron models with an M-type adaptation current that is associated with the Type II classification (Ermentrout et al., 2001).

We note that both neuron models contain sodium, delayed-rectifier potassium, and leak currents, while the Type II neuron with adaptation contains the additional slow potassium current. The difference between the Type I and Type II neuron models arises due to the different parameter values for the conductances and the differences in the functions governing the gating variables, primarily a depolarizing shift in the steady-state activation function associated with the delayed rectifier potassium channel. The difference between the Type I and Type II with adaptation neuron arises due to the activity of the slow potassium channel.

While the equations for the Type I neuron and the Type II neuron with adaptation were initially developed to model a cortical pyramidal neuron modulated by acetylcholine, the properties of this neuron when *g*_*K*_*s*__ = 0 closely mirror those of fast-spiking Type I interneurons (for instance, the PV interneurons modeled by Ferguson et al., 2013). Additionally, the presence of an active M-current when *g*_*K*_*s*__ = 1.5 causes this model to act similarly to interneurons with such a current, such as the OLM neurons.

For our network simulations, a constant, though heterogeneous between neurons, external input current is applied to all neurons, inducing continuous periodic firing which allows the PRC to be a useful tool for analyzing neural response properties. However, the PRC computed in response to a brief, weak input (as in Figures 1B,C) does not accurately describe the cell's response to the inhibitory synaptic input received within the network, because the synaptic transmission as modeled is not brief and weak. To understand how differences in intrinsic cellular properties affect responses to perturbations received by neurons within our model networks, we computed PRCs with an inhibitory signal approximating the magnitude and profile of the synaptic current received by a single cell following a burst of network activity. To differentiate PRCs calculated with this type of perturbation from those used to classify neuron type, we refer to the PRCs calculated with synaptic currents as sPRCs (as opposed to PRCs calculated with brief, excitatory current pulses which are referred to simply as PRCs). The sPRCs are shown in Figures 1D–F for Type I neurons, Type II neurons and Type II neurons with adaptation, respectively. For brief synaptic currents, sPRCs show similar properties to their PRC counterparts in Figure 1C, but as the duration of the synaptic current increases, the sPRCs for different cell types become more similar.

The sPRCs for the Type I neuron show a large delay response to perturbations delivered at early phases and exhibit linear properties, with a slope of approximately −1, as the phase of the perturbation increases. The magnitude of the delay depends upon the duration of the synaptic current. The linear properties of these sPRCs indicate that, regardless of timing, all perturbations serve to “reset” the neuron to the beginning of its firing cycle, where the neuron is held until the inhibition decays sufficiently. Furthermore, the neuron is held at the beginning of its firing cycle for the same duration regardless of when the perturbation occurred. The only factor that changes the magnitude of the phase delay, then, is the time elapsed between the initial action potential firing and the delivery of the perturbation. This evolves linearly with the timing of the perturbation. Since the scales of both the timing and the phase delay are normalized to 1, the sPRCs display linear properties with an approximate slope of −1. For these reasons, we classify sPRCs with linear characteristics as having “phase-resetting” properties.

The sPRCs for both the Type II neuron and the Type II neuron with adaptation exhibit a distinctly concave down shape for brief synaptic currents. As the duration of the synaptic current increases, the sPRCs for both of these neurons become more linear and start to resemble the phase-resetting shape. However, even for the longest lasting synaptic currents the sPRCs for the Type II neuron and the Type II neuron with adaptation still retain some concave down characteristics, never achieving the degree of linearity shown by the sPRCs for Type I neurons.

**Figure 2:**
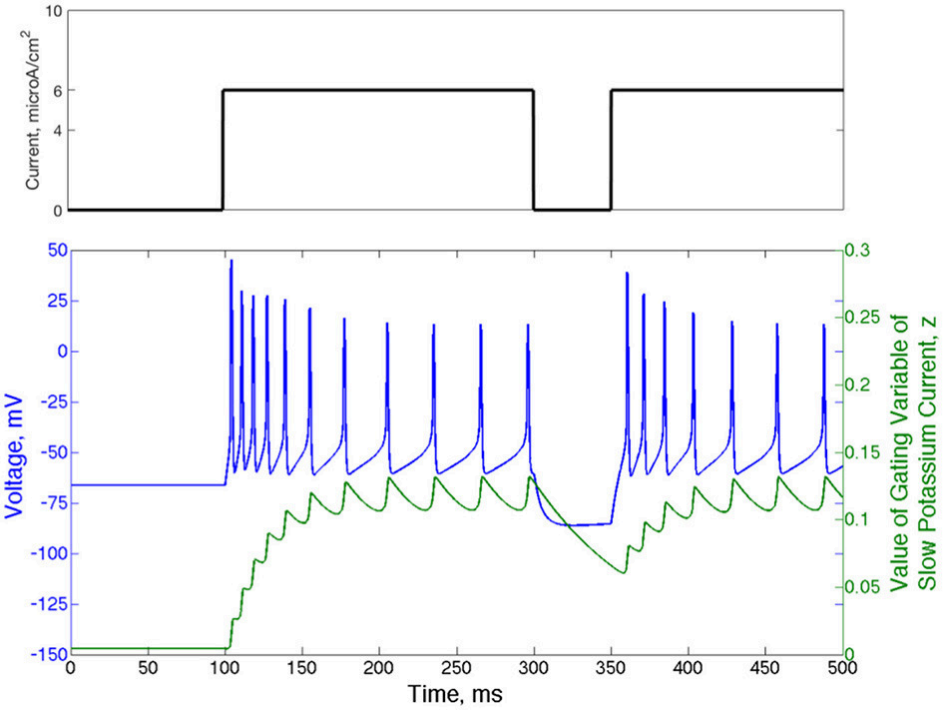
**Type II neurons with adaptation exhibit spike-frequency adaptation**. Voltage trace (blue) and value of the slow potassium gating variable *z* (green) shown for a single neuron that begins with no input current and equilibrium values of the voltage and all gating variables. The current step is shown above the voltage trace in black. The frequency of action potential firing depends upon the rate of previous action potential firing, which is reflected by the value of the gating variable of the slow potassium current.

Another relevant property of the Type II neuron with adaptation is spike-frequency adaptation. When this neuron is quiescent for a sufficient period of time, the gating variable governing the slow potassium current, *z*, falls below its typical value achieved during repetitive firing. When the neuron begins firing again, the slow time dynamics of *z* cause its value to rise slowly, allowing faster than normal firing until it fully recovers. These dynamics are displayed in Figure 2: in response to an applied current step initiated at *t* = 100 ms from resting membrane potential, action potential firing occurs at higher frequency until the value of *z* rises to a steady oscillation. Removal of the current step for a moderate period of time allows sufficient decay of *z* so that frequency is again high when the current step is reintroduced.

### 2.2. Network structure

We performed all simulations on networks of 1000 neurons. Each neuron received synaptic input from the same number (unless otherwise specified, 300) of randomly selected pre-synaptic cells.

Cell heterogeneity was implemented by varying the external input current, *I*_*app*_, to each neuron. The input currents were selected from a uniform distribution centered on the current (*I*_*A*_) that would impart an average intrinsic cell firing frequency to an isolated neuron. Here we studied networks with two levels of heterogeneity. For high heterogeneity simulations we chose the input currents uniformly from the distribution [0.9*I*_*A*_, 1.1*I*_*A*_], while for low heterogeneity simulations we chose the input currents uniformly from the distribution [0.99*I*_*A*_, 1.01*I*_*A*_].

We modeled synapses using a double exponential profile of the form

(18) $$\begin{array}{l} I_{syn}\left(t\right)=g_{syn}\left(V-E_{syn}\right)\left(\underset{\begin{array}{c} s_{i} \end{array}}{\sum }e^{-\left(t-s_{i}\right)∕\tau_{d}}-e^{-\left(t-s_{i}\right)∕\tau_{r}}\right) \end{array}$$

where *g*_*syn*_ is the maximum conductance, *V* is the membrane voltage of the post-synaptic neuron, *E*_*syn*_ is the reversal potential of the synapse, *s*_*i*_ are the times of all pre-synaptic spikes occuring before the current time *t* in ms, and τ_*d*_ and τ_*r*_ are the synaptic decay and the synaptic rise time constants, respectively (in ms). *E*_*syn*_ is set at −75 mV for inhibitory synapses. τ_*r*_ is set at 0.2 ms while τ_*d*_ is varied in the simulations. In all simulations, *g*_*syn*_ is set at 0.010 mS/cm^2^.

### 2.3. Measures

We used two measures to quantify the synchrony and clustering behavior of these networks. The Synchrony Measure is an adaptation of a measure created by Golomb et al. (Golomb and Rinzel, 1993, 1994) that quantifies the degree of spiking coincidence in the network. Briefly, the measure involves convolving a gaussian function with the time of each action potential for every cell to generate functions *V*_*i*_(*t*). The population averaged voltage *V*(*t*) is then defined as $V(t)=\frac{1}{N}\sum_{i=1}^{N}V_{i}(t)$, where *N* is the number of cells in the network, which for our simulations was 1000. We further define the overall variance of the population averaged voltage σ and the variance of an individual neuron's voltage σ_*i*_ as

(19) $$\begin{array}{l} \sigma =<V{\left(t\right)}^{2}>-<V\left(t\right){>}^{2} \end{array}$$

and

(20) $$\begin{array}{l} \sigma_{i}=<V_{i}{\left(t\right)}^{2}>-<V_{i}\left(t\right){>}^{2} \end{array}$$

where < ·> indicates time averaging over the interval for which the measure is taken. The Synchrony Measure *S* is then defined as

(21) $$\begin{array}{l} S=\frac{\sigma }{\frac{1}{N}\overset{N}{\underset{i=1}{\sum }}\sigma_{i}} \end{array}$$

The value *S* = 0 indicates completely asynchronous firing, while *S* = 1 corresponds to fully synchronous pattern of network activity.

To quantify relative overlap in cell participation in subsequent network bursts we constructed a new measure, entitled the Burst Similarity Measure. It quantifies the fraction of active neurons that participate in consecutive bursts of network activity. This measure is calculated in two steps. First, to detect timing and duration of each burst, the spike times of each neuron in the network are convolved with a gaussian function and a cumulative network activity trace is formed. This trace is subsequently thresholded to determine the on and off times for every burst (*b*_*j*_ and *e*_*j*_, respectively).

For each burst *j* we construct a binary vector that quantifies which neurons spiked during the burst, *v*_*j*_. If neuron *i* spiked during burst *j*, meaning it fires at a time *t*_*j*_ such that *b*_*j*_ ≤ *t*_*j*_ ≤ *e*_*j*_, we set *v*_*j*_(*i*) = 1, otherwise *v*_*j*_(*i*) = 0. The Burst Similarity Measure *B* is then determined via

(22) $$\begin{array}{l} B=\frac{1}{n-1}\overset{n-1}{\underset{j=1}{\sum }}\frac{v_{j}\cdot v_{j+1}}{\left|v_{j}||v_{j+1}\right|} \end{array}$$

where · indicates the vector dot product, |*x*| indicates the vector norm, and *n* is the total number of bursts.

A Burst Similarity Measure of *B* = 0 indicates that consecutive bursts contain mutually exclusive populations of neurons, while *B* = 1 indicates that consecutive bursts contain an identical population of neurons.

The measure allows for differentiation of multiple types of behavior reflected by an intermediate value of the Synchrony Measure. For example, one-cluster dynamics consisting of half the cells in the network being active, with the other half completely suppressed, exhibits an intermediate value of *S* and *B* = 1. In contrast, two-cluster dynamics consisting of bursts of network activity in which consecutive bursts contain mutually exclusive populations of neurons, each containing half of the neurons in the network, also has an intermediate value of *S* but *B* = 0.

Numerous validation studies were done to confirm that this measurement satisfies the above properties in practice. Toy cases easily confirm the extreme cases of *B* = 0 and *B* = 1 described above; additionally, for a variety of simulations of our networks we confirmed that the dynamics predicted by *B* matched the dynamics exhibited by the network by visually inspecting the corresponding raster plots.

### 2.4. Simulations

The code underlying these simulations was written in the C programming language and run on the University of Michigan's Flux cluster, a Linux-based high-performance computing cluster.

**Figure 3:**
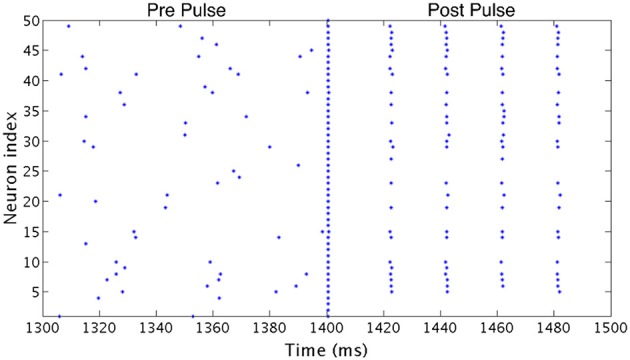
**Effect of a synchronizing current pulse in network simulations**. In an example network consisting of Type I neurons with high heterogeneity, a large, brief current pulse delivered at 1400 ms causes every cell in the network to fire synchronously. In response, this network changes behavior and exhibits one-cluster dynamics following the pulse despite firing asynchronously previously.

All simulations were run for 2500 ms from random initial conditions for voltage and gating variables for each neuron. Possible initial conditions for *V* ranged between −62 and −22 mv, while the possible initial conditions for each gating variable ranged between 0.2 and 0.8. In order to investigate the stability of the network's behavior, at a time of 1400 ms a large amplitude, brief current pulse was delivered to each cell in the network to cause all neurons to fire at the same time. As inhibitory synaptic currents do not directly promote synchronized firing and the ING mechanism achieves synchrony by organizing time windows that allow synchronized firing, this applied current pulse acts to impose an instance of synchrony on the network (analagous to imposing homogeneous initial conditions causing instantaneous spiking of all neurons in the network, as opposed to the randomized initial conditions that begin the simulations). To distinguish global convergence vs. local stability (relative to the entire state space of the model system) of synchronous and clustered solutions we applied the synchronizing pulse during the simulation and compared network dynamics established from random initial conditions (Pre Pulse) to that established after the current pulse (Post Pulse). An illustrative example of a clustered solution that is not globally convergent from random initial conditions but stable locally after the pulse is shown in Figure 3.

Model equations are integrated using a fourth order Runge-Kutta technique. Spikes do not trigger synaptic current until 100 ms into the simulation to allow initial transients to decay.

Color plots of the Synchrony Measure and the Burst Similarity Measure display the average of these scores over 10 independent simulations. The Pre Pulse scores (left panels) are calculated based on the network activity from 300 to 1300 ms, and the Post Pulse scores (right panels) are calculated based on the network activity from 1500 to 2500 s. Simulations (not shown here) were run to ensure that the behaviors indicated by the Synchrony Measure and Burst Similarity Measure taken over the given interval were indicative of stable behaviors that would persist long past the time interval measured here.

Color plots display measures for the same range of values for the synaptic decay time constant τ_*d*_, while the average intrinsic cell firing frequencies are chosen to sample a majority of the range of frequencies of repetitive cell firing that a given model can attain.

## 3. Results

We investigated global pattern formation in randomly connected inhibitory networks composed of neurons with three cellular excitability types and different levels of cellular heterogeneity, finding that the clustering dynamics were dependent upon cell type, heterogeneity level and the degree of connectivity. This diversity in network activity patterns provides evidence for the importance of intrinsic cell properties in dictating network patterns in randomly connected inhibitory networks, while also allowing for the identification of the mechanisms underlying these dynamics that depend upon these properties.

### 3.1. Effect of connectivity density

The computational study of neural networks includes a plethora of studies focusing on all-to-all connected networks. This literature includes many of the papers cited here as relevant to the study of interneuron networks, inhibitory networks, or the role of spike-frequency adaptation in network dynamics (Vreeswijk et al., 1994; Ermentrout et al., 2001; Goel and Ermentrout, 2002; Ermentrout and Wechselberger, 2009; Zahid and Skinner, 2009; Kilpatrick and Ermentrout, 2011; Dipoppa et al., 2012; Ladenbauer et al., 2012; Moon et al., 2015). One of the benefits of the study of all-to-all connected networks is the ability to use techniques, including weakly coupled oscillator theory and the phase-reduction technique, in order to mathematically analyze the network dynamics and in turn prove the generality of dynamical results. However, these techniques rely upon the assumption that the networks have all-to-all network topology (Schemer and Lewis, 2012).

**Figure 4:**
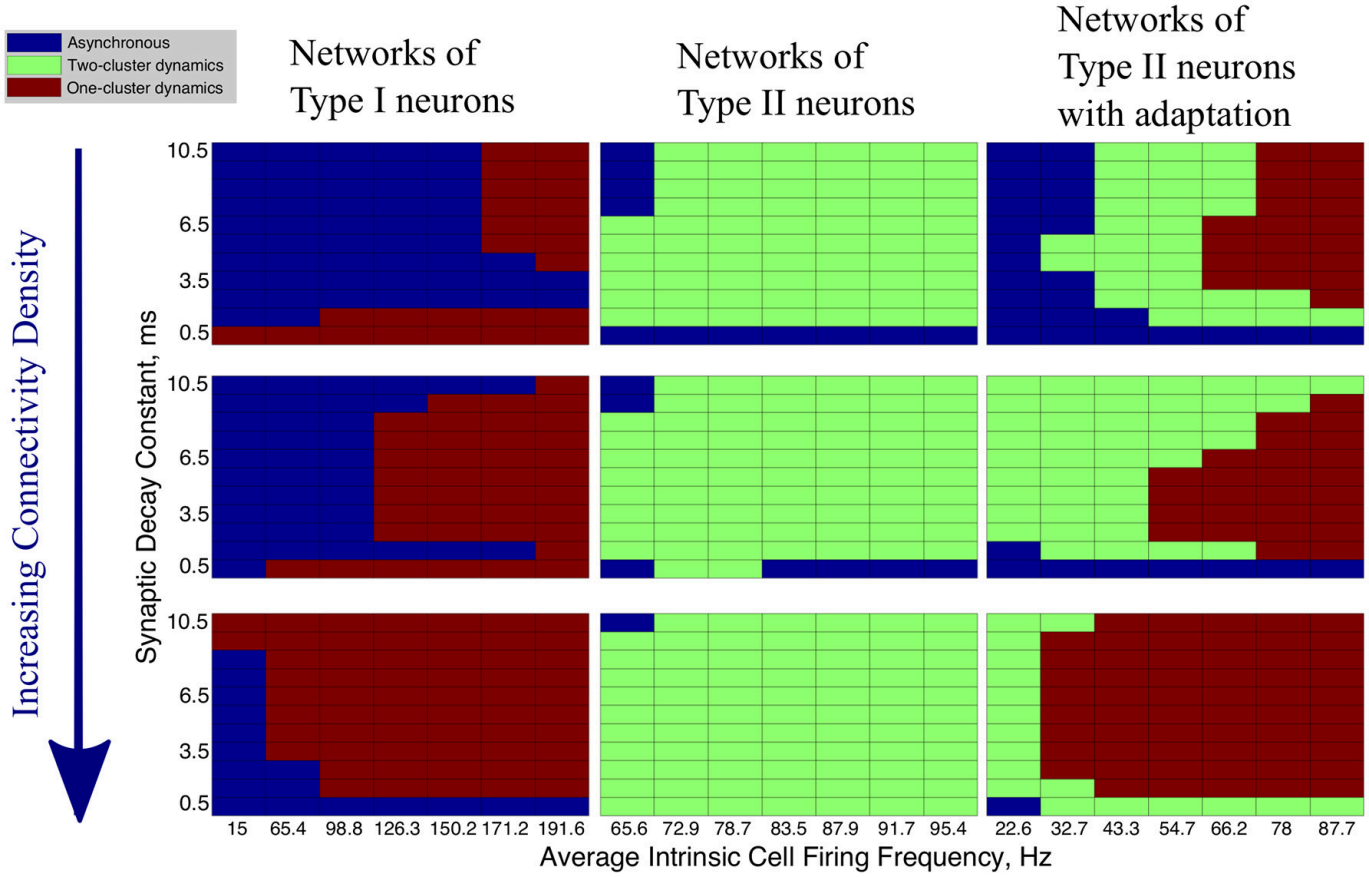
**Network activity patterns are dependent upon connectivity density**. Diagrams illustrating the changing network dynamics in our simulations as a function of connectivity density and neuron type, with simulations run with a range of average intrinsic cell firing frequencies (horizontal axis) and synaptic decay constants (vertical axis). The connectivity densities shown here are 10, 30, and 100%, from top to bottom. Simulations for Type I neurons are shown in the first column, simulations for Type II neurons are shown in the second column, and simulations for Type II neurons with adaptation are shown in the third column.

In our networks with high heterogeneity in intrinsic cell firing frequency, the level of connectivity density caused significant changes in the patterns of network dynamics. These changes are shown by the changing network dynamics illustrated in Figure 4 for networks of Type I (first column), Type II (second column) and Type II with adaptation (third column) neurons, for networks with a connectivity density of 10, 30, and 100%, from top to bottom, respectively.

The values of the Synchrony Measure (*S*) and Burst Similarity Measure (*B*), when analyzed jointly, indicate the type of activity in these networks and inform the classification of network dynamics in Figure 4. We describe the manner in which *S* and *B* are analyzed to yield the classification of network dynamics below. For simplicity, in Figure 4 we only illustrate the changes in overall network dynamics.

Regardless of the connectivity density, synchronous activity in networks of Type I neurons was restricted to one-cluster dynamics. The parameter space in which one-cluster firing occurred, as opposed to asynchronous dynamics, moved to include lower intrinsic cell firing frequencies as the connectivity density increased. While networks with 30% connectivity density do not evolve to one-cluster dynamics from randomized initial conditions at low intrinsic firing frequencies, as shown here, we will show below that these networks can achieve one-cluster dynamics following the synchronizing current pulse.

When connectivity density was less than 30%, networks of Type II neurons with adaptation exhibited one-cluster dynamics for high average intrinsic cell firing frequencies, but displayed two-cluster dynamics or asynchronous activity as cell firing frequency decreased or the synaptic decay time constant increased. As the connectivity density increased, parameter regimes which supported two-cluster dynamics at lower connectivity densities exhibited one-cluster dynamics at the higher connectivity densities. At full connectivity density, two-cluster dynamics were only found for networks with the slowest average cell firing frequencies or shortest synaptic decay constants. Thus, in these networks, lower connectivity density allows cellular and synaptic properties to influence network activity and determine whether two-cluster dynamics or one-cluster dynamics are exhibited. In fully connected networks, cellular and synaptic properties are less influential and network dynamics converge to similar patterns of one-cluster dynamics.

Connectivity density had minimal effect on the type of synchronous dynamics exhibited in networks of Type II neurons. For all densities, networks displayed two-cluster dynamics with little effect due to variations in intrinsic cell firing frequency and synaptic decay time constant.

In summary, increasing connectivity density limited the contributions of cellular and synaptic properties to network dynamics in our simulations. Not surprisingly, full connectivity promoted one-cluster synchronous dynamics, except in the Type II networks. We note, though, that when heterogeneity in intrinsic cell firing frequency was reduced in Type II networks, one-cluster dynamics were exhibited (see further results below).

For the remainder of this study, we consider networks with 30% connectivity density. There exists biological evidence for 30% connectivity density among inhibitory neurons based on data from the CA1 region of the rat hippocampus (Ascoli and Atkeson, 2005; Viriyopase et al., 2016). Additionally, from the above results, networks with this connectivity showed distinct dynamical patterns from both extremely sparsely and extremely densely connected networks, thus making generalizations from the study of these networks to other randomly connected networks reasonable.

### 3.2. Networks of type I neurons

**Figure 5:**
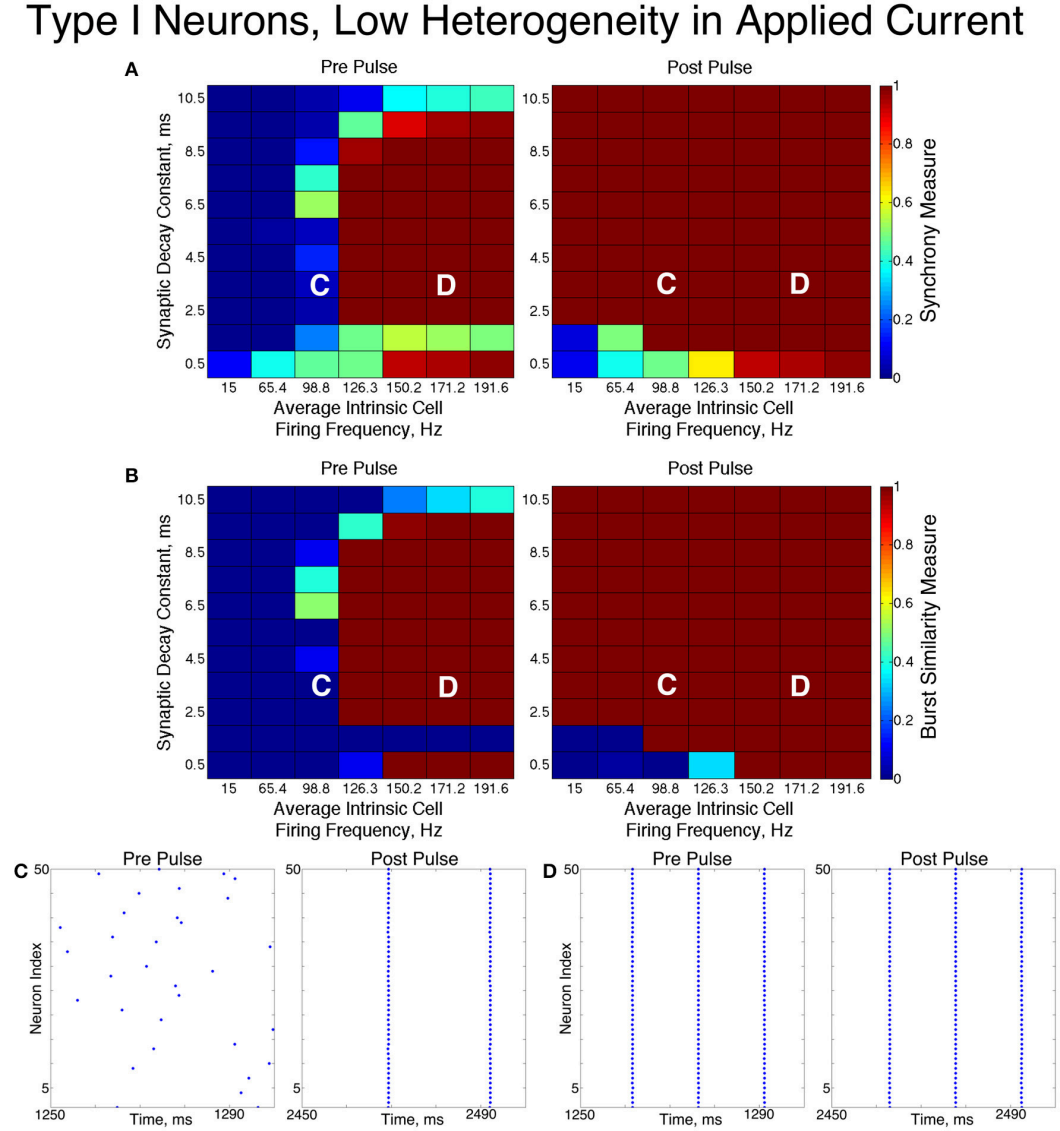
**Dynamics of networks of Type I neurons with low cellular heterogeneity**. **(A,B)** Synchrony Measure **(A)** and Burst Similarity Measure **(B)** for simulations run with a range of average intrinsic cell firing frequencies (horizontal axis) and synaptic decay constants (vertical axis), averaged over 10 independent simulations before (left panel) and after (right panel) the synchronizing current pulse. **(C)** Example raster plot for a simulation with an average intrinsic cell firing frequency of 98.8 Hz and a synaptic decay constant of 3.5 ms (whose position in the heatmaps is illustrated by the overlayed C) shows asynchrony occuring from initial conditions but full synchrony following the pulse. **(D)** Example raster plot for a simulation with an average intrinsic cell firing frequency of 171.2 Hz and a synaptic decay constant of 3.5 ms (whose position in the heatmaps is illustrated by the overlayed D) exhibits full synchrony before and after the pulse.

**Figure 6:**
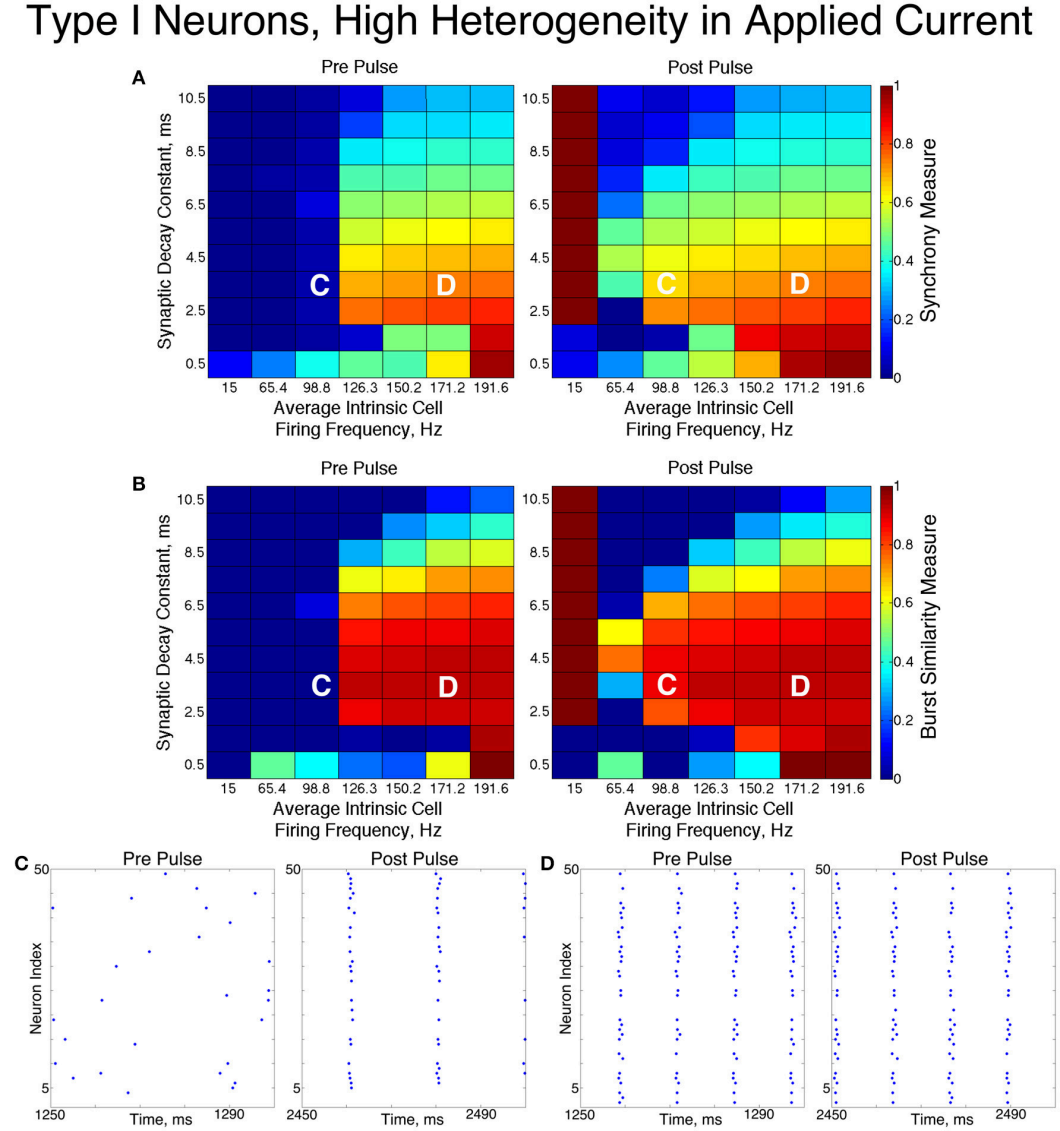
**Dynamics of networks of Type I neurons with high cellular heterogeneity**. **(A,B)** Synchrony Measure **(A)** and Burst Similarity Measure **(B)** for simulations run with a range of average intrinsic cell firing frequencies (horizontal axis) and synaptic decay constants (vertical axis), averaged over 10 independent simulations before (left panel) and after (right panel) the synchronizing current pulse. **(C)** Example raster plot for a simulation with an average intrinsic cell firing frequency of 98.8 Hz and a synaptic decay constant of 3.5 ms (whose position in the heatmaps is illustrated by the overlayed C) shows asynchrony occuring from initial conditions but one-cluster dynamics following the pulse. **(D)** Example raster plot for a simulation with an average intrinsic cell firing frequency of 171.2 Hz and and a synaptic decay constant of 3.5 ms (whose position in the heatmaps is illustrated by the overlayed D) exhibits one-cluster dynamics before and after the pulse.

Inhibitory networks of Type I neurons manifested full synchrony, one-cluster dynamics, or asynchrony with 30% connectivity density. The particular type of behavior exhibited by the network was determined by the two parameters varied across simulations, the average intrinsic cell firing frequency of neurons in the network and the synaptic decay time constant, as well as the level of heterogeneity. Full synchrony was exhibited only when the level of heterogeneity was low (Figure 5); in this case the network exhibited bursts of network activity containing every neuron in the network. In the high heterogeneity case (Figure 6), one-cluster dynamics were observed in which bursts contained largely the same group of neurons but not all neurons in the network, which is indicative of cell suppression (Chow et al., 1998).

The dynamics exhibited by the network were determined by the values of *S* and *B*. High values of both measures indicate that the network exhibited one-cluster dynamics: high values of *S* indicate that some clustering occurred in the network, and high values of *B* indicate that subsequent bursts of network activity contained similar populations of neurons. After inspecting the values of both *S* and *B* corresponding with various network behaviors and visually classifying dynamics in the corresponding raster plots, we determined that clustering occurs when *S* > 0.4, and when *B* > 0.2 subsequent bursts are sufficiently similar for the behavior to be deemed one-cluster dynamics (although the values of *B* observed for one-cluster dynamics were typically much higher than this level). Both measures approaching their maximal value of 1 indicated that the network exhibits full synchrony. Two-cluster dynamics are indicated by a moderate value of *S* (typically *S*≈0.6), but *B* = 0.

The stability of the solutions observed from randomized initial conditions were investigated by applying a synchronizing current pulse to all neurons approximately midway through the simulation. The current pulse caused all neurons to fire simultaneously which produced a subsequent uniform suppression of all neurons via the synaptic inhibition. The left and right panels of Figures 5, 6 display the measures and example raster plots of pre- and post-pulse network activity, respectively.

Networks with low average intrinsic cell firing frequencies exhibited asynchronous activity patterns, regardless of heterogeneity and synaptic decay time constant, when simulated from random initial conditions. However, stable full synchrony or one-cluster dynamics could be induced by the synchronizing current pulse in some of these networks: in nearly all networks with low heterogeneity, the current pulse induced full synchrony, while in networks with high heterogeneity the pulse induced one-cluster dynamics within a range of synaptic decay time constant values. Thus, the current pulse revealed bistable dynamics in these networks characterized by evolution to either asynchronous or clustered dynamics depending on initial conditions. Full synchrony developed following the current pulse in networks with high heterogeneity and an average intrinsic cell firing frequency of 15 because *I*_*A*_ for these networks was 0, resulting in networks with homogeneous external input current to the neurons. Examples of these bistabilities between asynchronous and fully synchronous or one-cluster firing are shown in the raster plots in Figures 5C, 6C.

This bistability is reflective of properties of the ING mechanism. Asynchrony occurred due to the combination of the low firing frequency of neurons and the presence of synaptic inhibition preventing enough cells from firing in close enough temporal proximity to generate sufficient synaptic inhibition to suppress spiking activity in the entire network for a sufficient period. However, the current pulse instantiated a state in which every neuron in the network fired synchronously; following this, every neuron in the network received identically strong synaptic inhibition, initiating ING-driven activity. Following the pulse, this synchronous activity was fully maintained in most of the low heterogeneity networks, and partially preserved via one-cluster dynamics in many high heterogeneity networks.

**Figure 7:**
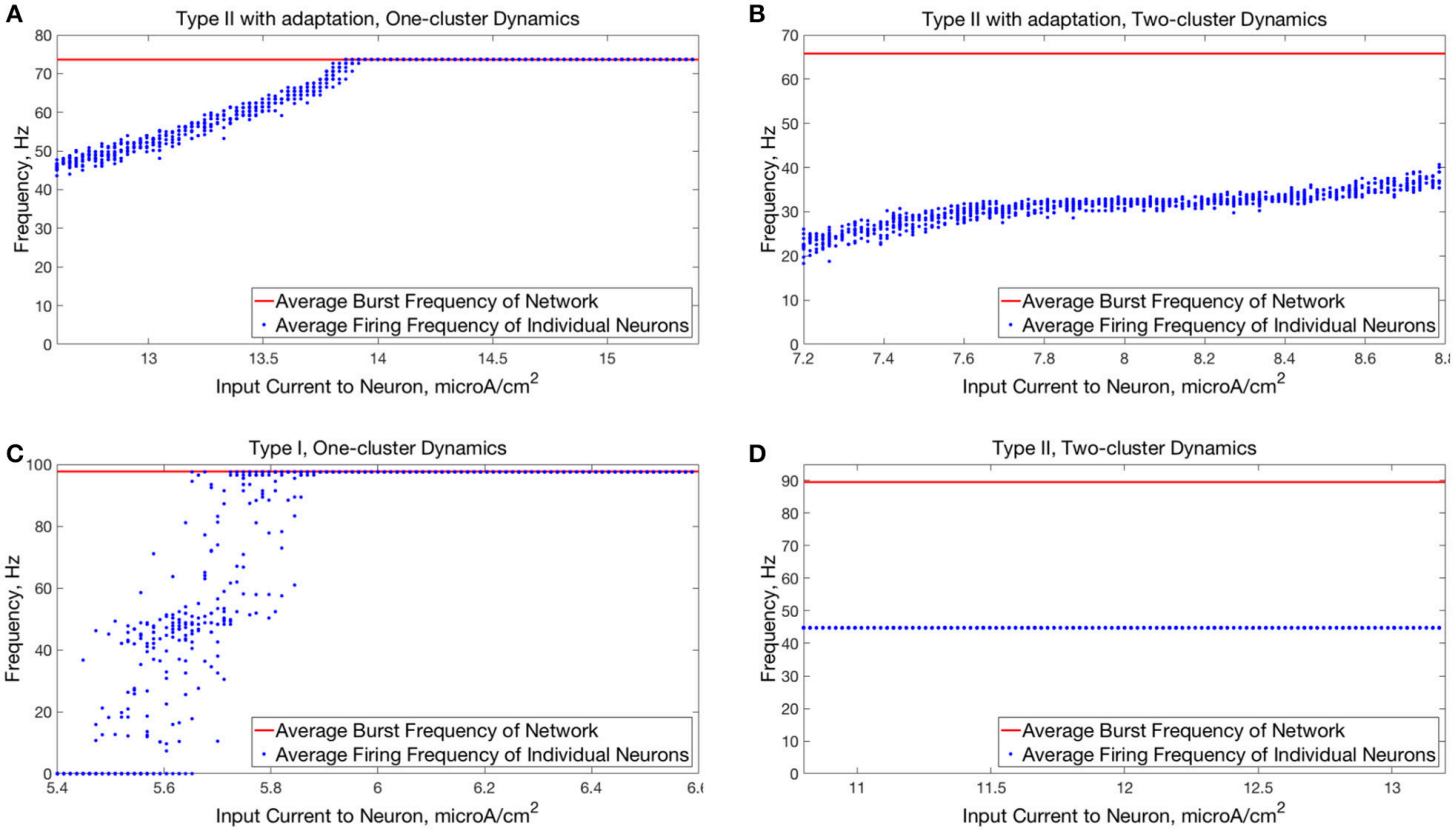
**Comparison of relationships between input current and average neuron firing frequencies in one-cluster and two-cluster network dynamics**. **(A)** Average firing frequencies of individual neurons in a network of Type II neurons with adaptation exhibiting one-cluster dynamics plotted against the input current to the corresponding neuron. **(B)** Same as **(A)** but for a network of Type II neurons with adaptation exhibiting two-cluster dynamics. **(C)** Same as **(A)** but for a network of Type I neurons with similar values of *S* and *B* as in **(A)**. **(D)** Same as **(B)** but for a network of Type II neurons with similar values of *S* and *B* as in **(B)**.

Networks with higher average intrinsic cell firing frequencies showed no significant changes in either *S* or *B* after the current pulse, indicating that ING-driven dynamics are globally stable solutions that do not depend on initial conditions. These networks exhibited full synchrony in the low heterogeneity case and one-cluster dynamics in the high heterogeneity case. Example raster plots in this domain, illustrating the similarity in network activity before and after the current pulse, are shown in Figures 5D, 6D. In the high heterogeneity case, the range of computed *S* values reflect the proportion of cells in the network that participate in the one-cluster dynamics, with lower values of *S* indicating that fewer cells participated in each burst of network activity. In networks with a lower *S*, neurons that have smaller input currents either participated in very few bursts or were completely suppressed. This is illustrated by Figure 7C, which shows the relationship between input current and average firing frequency for each individual neuron in an example network illustrating cell suppression. These results match previous results studying analagous networks with heterogeneity (Wang and Buzsáki, 1996; Chow et al., 1998; Bartos et al., 2002; Tiesinga and Sejnowski, 2009; Ferguson et al., 2013).

The sPRCs shown in Figure 1D help to explain these networks' tendency to exhibit dynamics driven by the ING mechanism. Regardless of the duration of the synaptic current, the sPRCs for Type I neurons showed strong phase-resetting characteristics. As discussed above, phase-resetting characteristics arise in an sPRC when the synaptic inhibition holds a cell at the beginning of its firing cycle for the duration of the synapse, irregardless of the signal's timing. When all the cells in the network receive this type of perturbation, they become suppressed until the synaptic signal decays, eliciting a “window” in which cell firing can occur before the next round of action potential firing and the resulting synaptic inhibition suppresses the neurons once again. This is exactly the underlying mechanism of ING, indicating that the tendency for the ING-driven dynamics in these networks can be explained by the phase-resetting characteristics of the sPRC.

Thus, our results in this case agreed with the theory of the ING mechanism, as our networks exhibited full synchrony or one-cluster dynamics with the degree of synchrony within these clusters dependent upon heterogeneity, the synaptic decay constant, and the average intrinsic cell firing frequency of neurons in the network. Further evidence for the ING mechanism driving the synchronous activity was in the response of these networks to the current pulse that artificially instantiated synchronous dynamics into these networks.

### 3.3. Networks of type II neurons

The networks of Type II neurons typically exhibited either full synchrony or two-cluster dynamics. The type of behavior displayed depended upon the synaptic decay time constant and level of cellular heterogeneity. Interestingly, for the range of synaptic decay time constants and average intrinsic cell firing frequencies studied here, networks of Type II neurons only exhibited ING-driven full synchrony in the low heterogeneity case. This provides strong evidence that intrinsic cellular properties are important in determining clustering dynamics in networks with non-trivial levels of heterogeneity, which are more biologically plausible than homogeneous networks.

**Figure 8:**
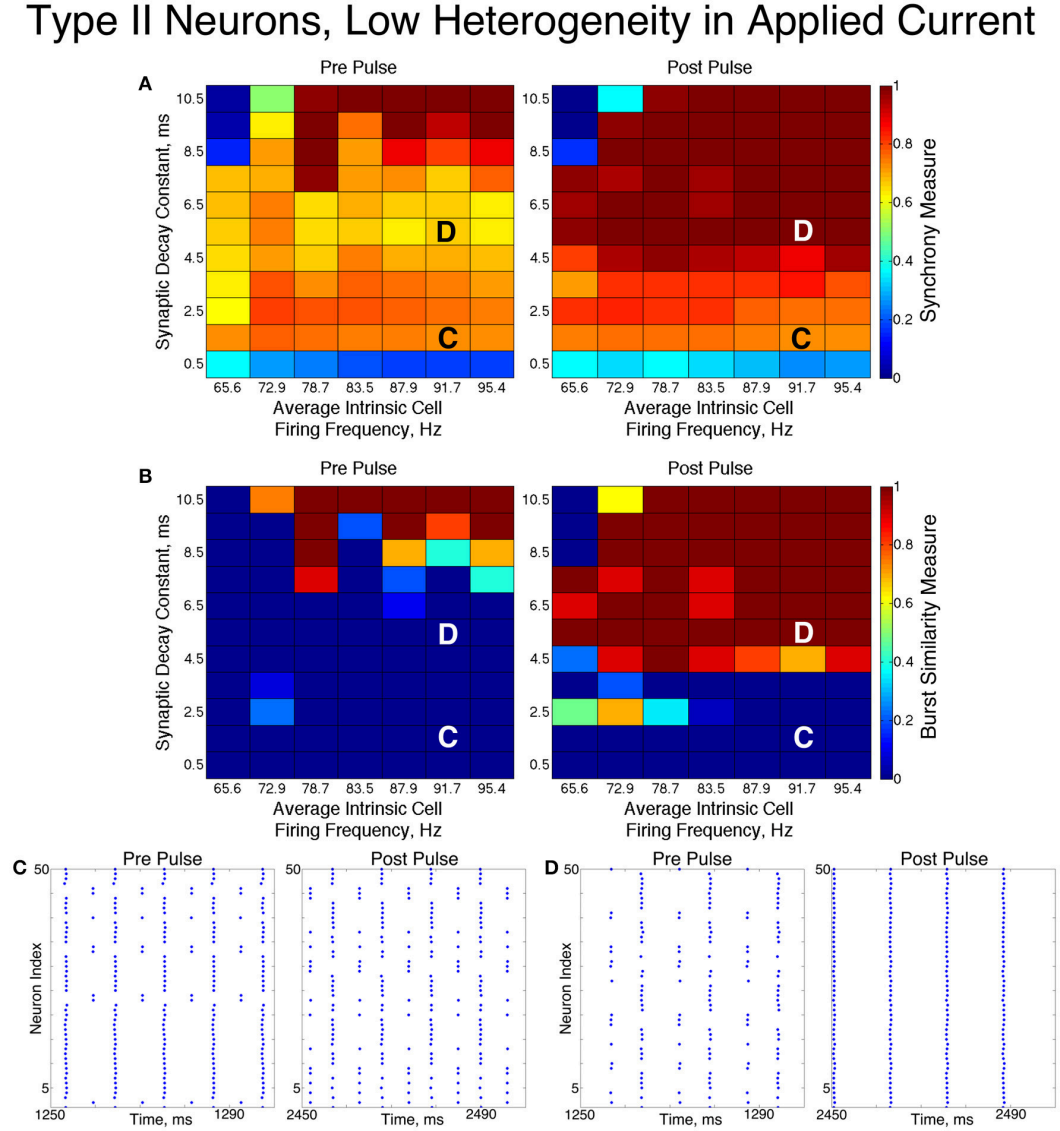
**Dynamics of networks of Type II neurons with low cellular heterogeneity**. **(A,B)** Synchrony Measure **(A)** and Burst Similarity Measure **(B)** for simulations run with a range of average intrinsic cell firing frequencies (horizontal axis) and synaptic decay constants (vertical axis), averaged over 10 independent simulations before (left panel) and after (right panel) the synchronizing current pulse. **(C)** Example raster plot for a simulation with an average intrinsic cell firing frequency of 91.7 Hz and a synaptic decay constant of 1.5 ms (whose position in the heatmaps is illustrated by the overlayed C) exhibits two-cluster dynamics before and after the synchronizing current pulse. **(D)** Example raster plot for a simulation with an average intrinsic cell firing frequency of 91.7 Hz and and a synaptic decay constant of 5.5 ms (whose position in the heatmaps is illustrated by the overlayed D) exhibits two-cluster dynamics before the pulse but full synchrony after the pulse.

**Figure 9:**
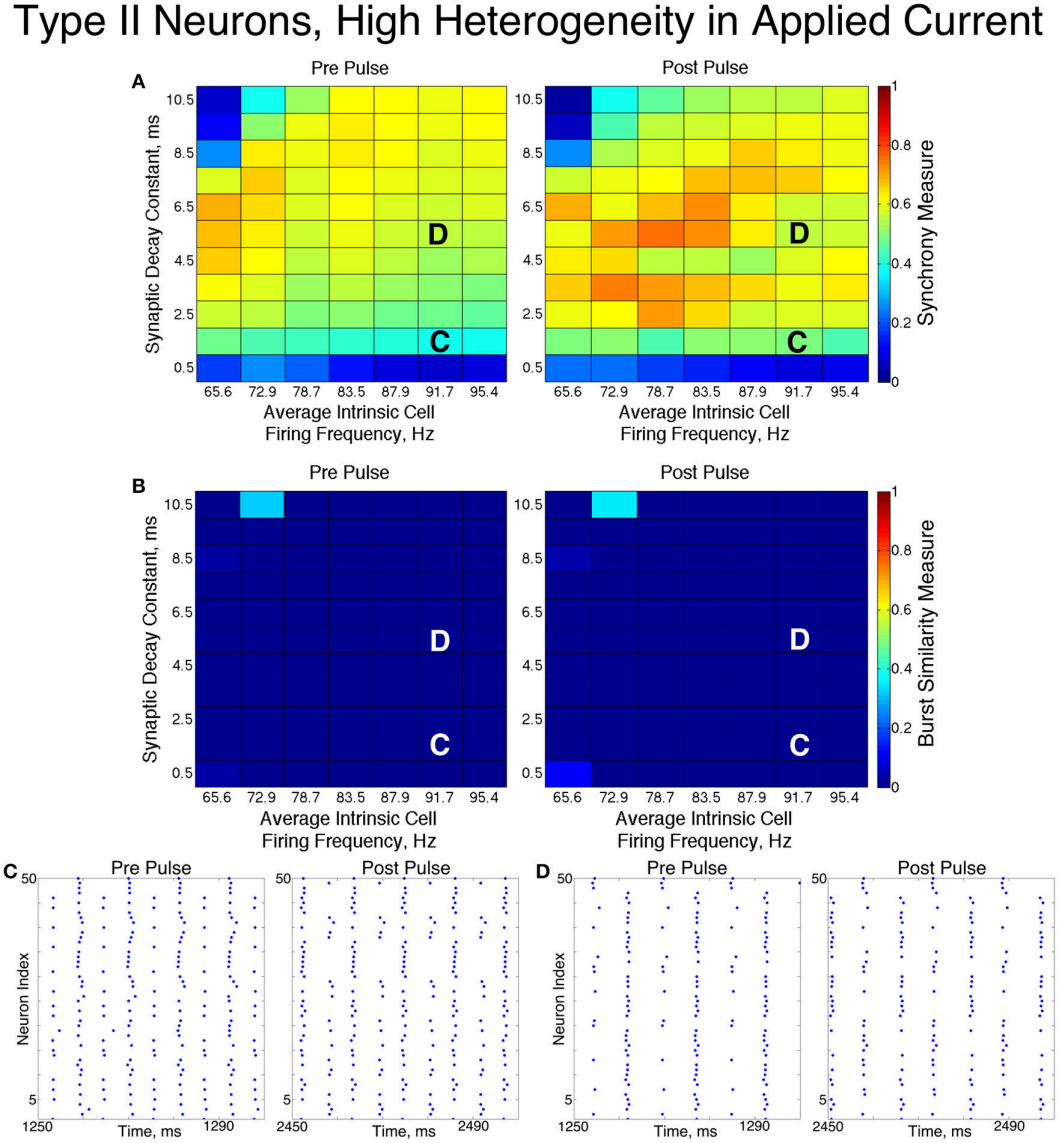
**Dynamics of networks of Type II neurons with high cellular heterogeneity**. **(A,B)** Synchrony Measure **(A)** and Burst Similarity Measure **(B)** for simulations run with a range of average intrinsic cell firing frequencies (horizontal axis) and synaptic decay constants (vertical axis), averaged over 10 independent simulations before (left panel) and after (right panel) the synchronizing current pulse. **(C)** Example raster plot for a simulation with an average intrinsic cell firing frequency of 91.7 Hz and a synaptic decay constant of 1.5 ms (whose position in the heatmaps is illustrated by the overlayed C) exhibits two-cluster dynamics before and after the synchronizing current pulse. **(D)** Example raster plot for a simulation with an average intrinsic cell firing frequency of 91.7 Hz and a synaptic decay constant of 5.5 ms (whose position in the heatmaps is illustrated by the overlayed D) exhibits two-cluster dynamics before and after the synchronizing current pulse.

For low values of the synaptic decay time constant, networks with low heterogeneity (Figure 8) exhibited two-cluster dynamics. When the decay constant was large, the network evolved to full synchrony from random initial conditions, but for moderate values of the decay constant the synchronizing current pulse was necessary to induce full synchrony. Thus, in this regime the network displayed bistability between a two-cluster solution and full synchrony. Networks of Type II neurons with high heterogeneity (Figure 9) almost exclusively exhibited two-cluster dynamics. The current pulse did not induce full synchrony or one-cluster dynamics, but instead the synchronous firing instantiated by the pulse was not maintained and firing evolved back into two distinct clusters.

The fact that low heterogeneity networks showed a response to the current pulse, while high heterogeneity networks did not, implies that ING-driven synchrony plays a role in networks of Type II neurons when the heterogeneity is sufficiently low, but a different mechanism controls behavior in the high heterogeneity case.

**Figure 10:**
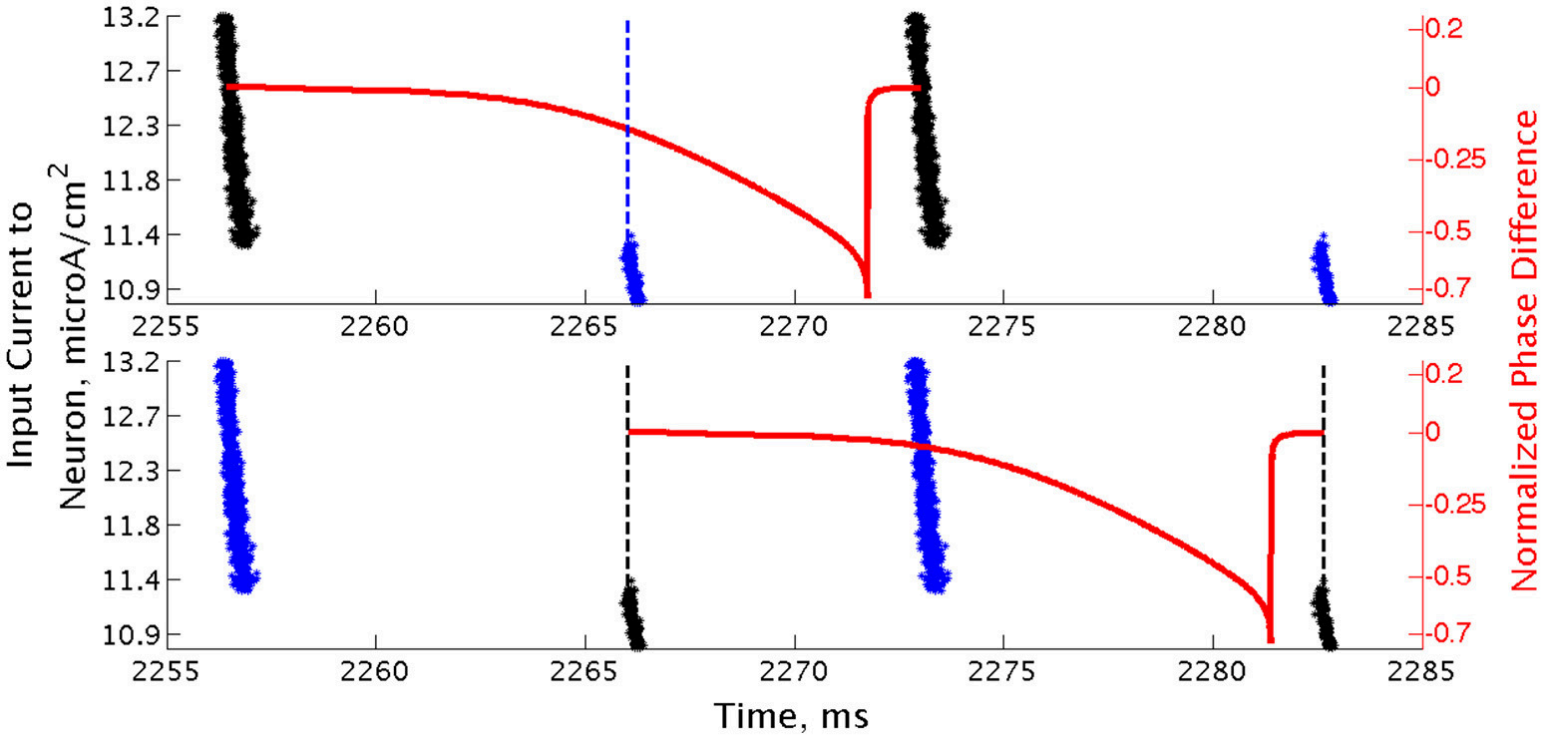
**Clusters in networks of Type II neurons are segregated based upon neurons' intrinsic firing frequency**. Raster plot of a high heterogeneity Type II network with an average intrinsic cell firing frequency of 72.9 Hz and a synaptic decay constant of 3.5 ms, with neurons organized based upon their external input current. Overlaid with this plot is a sPRC, generated from analogous synaptic parameters for the neuron firing at a similar frequency to those in the network, showing the timing of firings of the clusters relative to each other (dashed lines added at the beginning of bursts to emphasize these timings). While the raster plots in the bottom and top panels are identical, the overlaid sPRC begins with the black burst in each panel in order to emphasize the timing differences in the cluster firings relative to the effect articulated by the sPRC.

Closer analysis of networks of Type II neurons exhibiting two-cluster dynamics showed that the two clusters were easily differentiated: one cluster consisted of neurons with smaller external input currents (*I*_*app*_) and thus lower intrinsic firing frequencies, while the other cluster consisted of neurons with higher input currents and associated intrinsic firing frequencies. Furthermore, the timing of cluster firings was asymmetric. The cluster containing neurons with lower *I*_*app*_ values typically fired later in the cycle between firings of the cluster containing neurons with higher *I*_*app*_ values, while the cluster containing neurons with higher *I*_*app*_ values typically fired earlier in the cycle between firings of the low *I*_*app*_ cluster (Figure 10).

Properties of the sPRC for Type II neurons explain this phenomenon. When calculated using an inhibitory perturbation matching the synaptic current, the sPRC (red curve in Figure 10) is approximately flat for perturbations arriving early in a neuron's firing cycle, but the response begins to change rapidly as the timing of the perturbation occurs later in the neuron's period. As the perturbation to the cells in the high *I*_*app*_ cluster from the firing of the low *I*_*app*_ cluster occurred at later phases in their firing cycle, these faster firing cells responded with a larger phase delay (top panel). In contrast, since the perturbation to the cells in the low *I*_*app*_ cluster from the firing of the high *I*_*app*_ cluster occurred at earlier phases, the induced phase delay to these slower firing cells was smaller (bottom panel). This difference in the magnitude of phase delays induced in the two clusters served to balance the frequency differences among their constituent cells, and in turn organized network activity into stable two-cluster dynamics. The properties of the sPRC that underlie this mechanism are distinct from the phase-resetting properties displayed by Type I neuron sPRCs.

This hypothesis was further supported by the timing of firing of cells within each cluster. Within the burst firing of the high *I*_*app*_ cluster (the larger cluster in Figure 10), the neurons with the highest *I*_*app*_ values fired earliest, thus responding with a greater phase delay than the neurons firing later within the cluster. This pattern of cell firing within the cluster balances the effect of the heterogeneity in external input current. This feature holds true for the timing of cell firing within the low *I*_*app*_ cluster as well.

The general shape and skew properties of the sPRC shown in Figure 10 are present for Type II neuron sPRCs with all but the largest synaptic decay time constants, as shown in Figure 1E. Only for the longest lasting synaptic currents do these properties diminish and phase-resetting properties appear. This helps to explain why for longer lasting synaptic currents, networks with low heterogeneity displayed full synchrony analagous to that seen in similar networks of Type I neurons. The diminished phase-resetting characteristics of the sPRCs calculated for Type II neurons imply that cellular properties play a more important role in determining the dynamics of networks of these neurons as opposed to networks of Type I neurons.

### 3.4. Networks of type II neurons with an M-Type adaptation current

**Figure 11:**
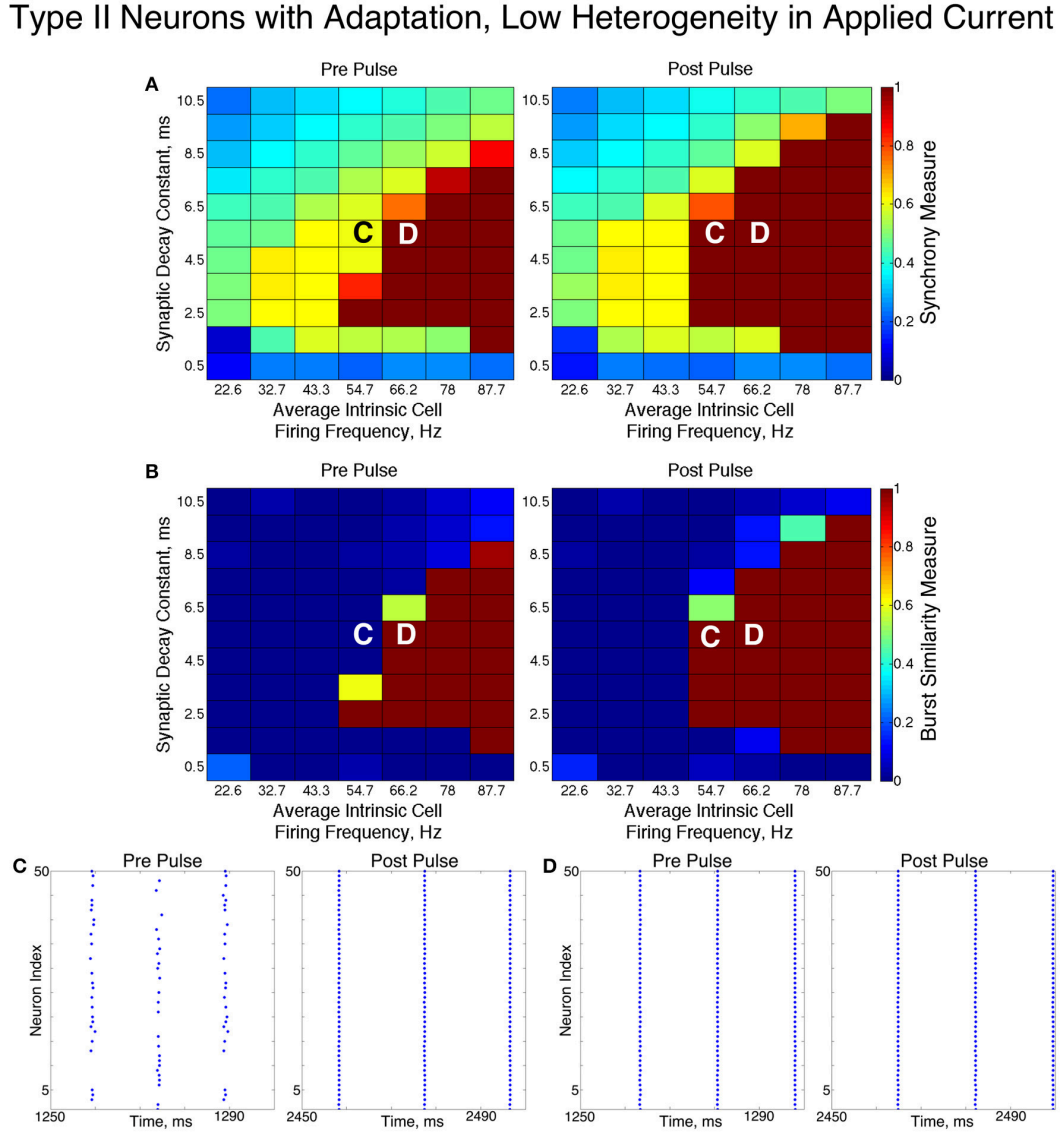
**Dynamics of networks of Type II neurons with adaptation with low cellular heterogeneity**. **(A,B)** Synchrony Measure **(A)** and Burst Similarity Measure **(B)** for simulations run with a range of average intrinsic cell firing frequencies (horizontal axis) and synaptic decay constants (vertical axis), averaged over 10 independent simulations before (left panel) and after (right panel) the synchronizing current pulse. **(C)** Example raster plot for a simulation with an average intrinsic cell firing frequency of 54.7 Hz and a synaptic decay constant of 5.5 ms (whose position in the heatmaps is illustrated by the overlayed C) exhibits two-cluster dynamics prior to the pulse but full synchrony following the pulse. **(D)** Example raster plot for a simulation with an average intrinsic cell firing frequency of 66.2 Hz and and a synaptic decay constant of 5.5 ms (whose position in the heatmaps is illustrated by the overlayed D) exhibits full synchrony both before and after the pulse.

We found that networks of Type II neurons with adaptation exhibited all considered types of dynamics: asynchrony, full synchrony, one-cluster and two-cluster dynamics. The exhibited spatio-temporal pattern depended upon the average intrinsic cell firing frequency and synaptic decay time constant. In the low heterogeneity case (Figure 11), for higher intrinsic cell firing frequencies there was a bounded range of synaptic decay constant values that led to full synchrony. Outside of this regime, two-cluster dynamics were primarily observed, with a small region of asynchronous behavior for very brief synaptic currents. Networks primarily evolved to these dynamics from random initial conditions and the synchronizing current pulse revealed minimal regions of bistability.

**Figure 12:**
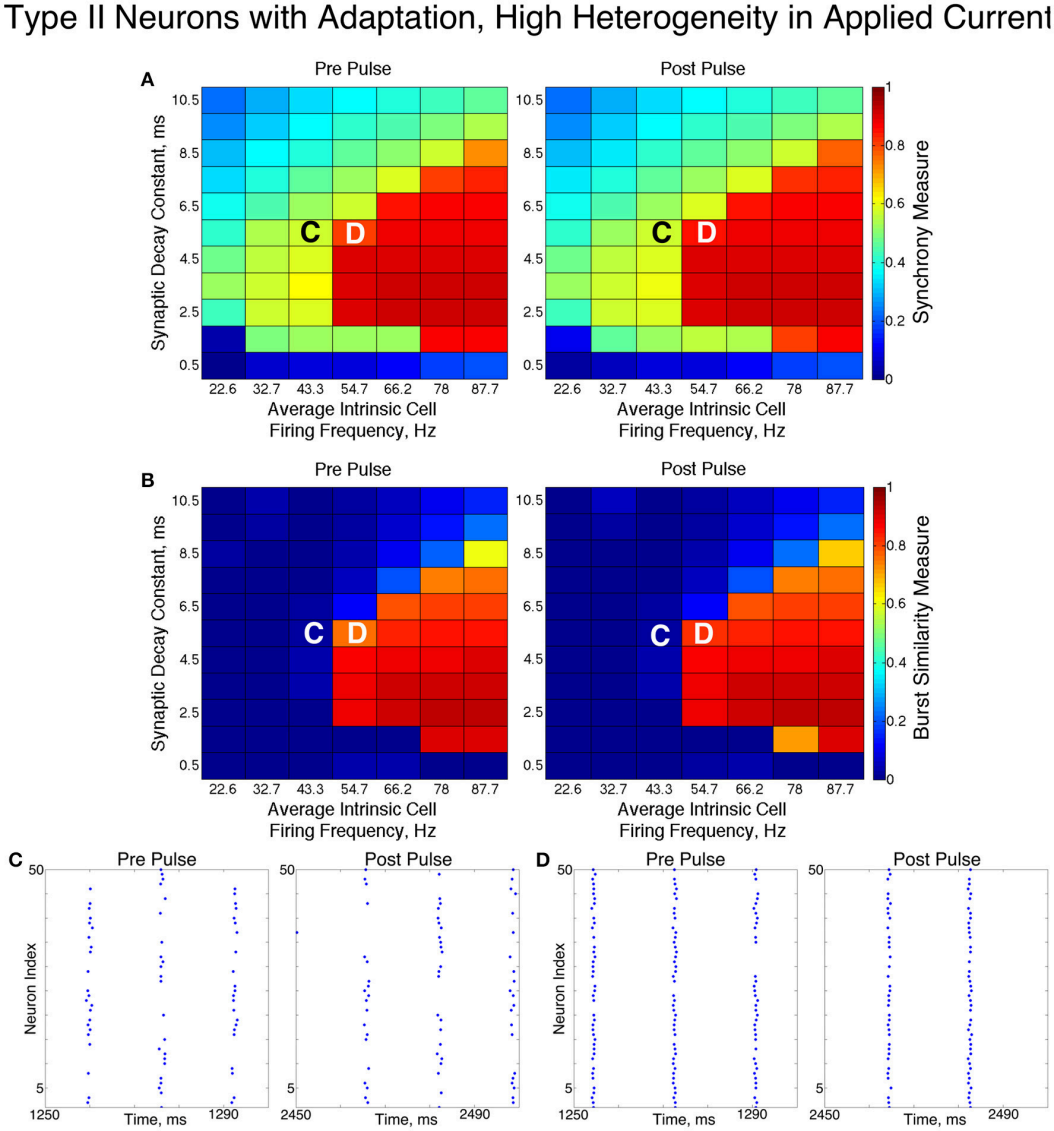
**Dynamics of networks of Type II neurons with adaptation with high cellular heterogeneity**. **(A,B)** Synchrony Measure **(A)** and Burst Similarity Measure **(B)** for simulations run with a range of average intrinsic cell firing frequencies (horizontal axis) and synaptic decay constants (vertical axis), averaged over 10 independent simulations before (left panel) and after (right panel) the synchronizing current pulse. **(C)** Example raster plot for a simulation with an average intrinsic cell firing frequency of 43.3 Hz and a synaptic decay constant of 5.5 ms (whose position in the heatmaps is illustrated by the overlayed C) exhibits two-cluster dynamics both before and after the synchronizing current pulse. **(D)** Example raster plot for a simulation with an average intrinsic cell firing frequency of 54.7 Hz and and a synaptic decay constant of 5.5 ms (whose position in the heatmaps is illustrated by the overlayed D) exhibits one-cluster dynamics both before and after the synchronizing current pulse.

In these networks, dynamics were robust to cellular heterogeneity. The high heterogeneity case (Figure 12) showed very similar results as those observed in the low heterogeneity case, although the region of full synchrony was replaced by a region of one-cluster dynamics in which not every cell participated in each burst. No bistability between two-cluster and one-cluster firing was observed in response to the synchronizing current pulse for high heterogeneity networks.

For both high and low heterogeneity simulations, in the regime in which two-cluster dynamics was observed the synchronizing current pulse had minimal effect. Because the effect of the current pulse is to induce ING-driven full synchrony or one-cluster dynamics where possible, this led us to conclude that ING-driven synchrony is not achievable in a large majority of the networks that exhibited two-cluster dynamics.

**Figure 13:**
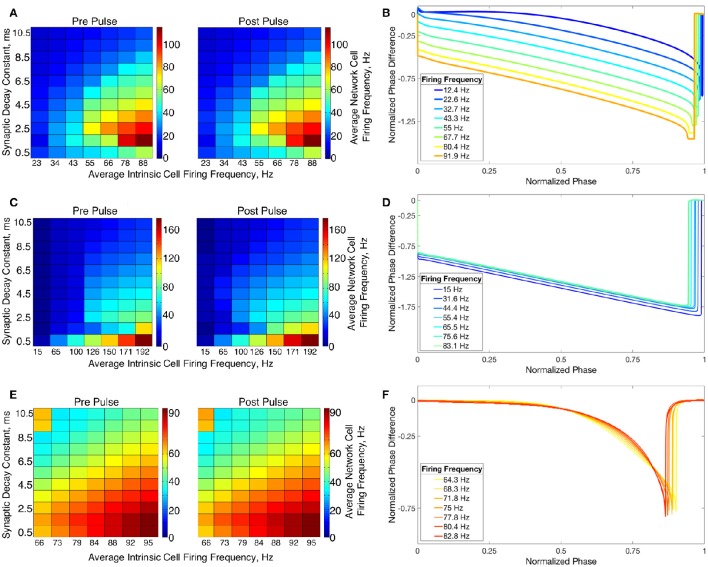
**Relationship between average network cell firing frequency and network dynamics is explained by properties of corresponding sPRCs**. **(A)** Average firing frequency of neurons in networks of Type II neurons with adaptation in the high heterogeneity case, both before and after the current pulse and averaged over ten independent simulations. **(B)** sPRCs for a Type II neuron with adaptation naturally firing at various frequencies, calculated with a double exponential synaptic current perturbation with a synaptic decay constant of 3.5 ms. **(C–F)** Same as **(A,B)** but for Type I neurons **(C,D)** and Type II neurons **(E,F)**.

The transition from two-cluster dynamics to one-cluster dynamics in the high heterogeneity case, or to full synchrony in the low heterogeneity case, as average intrinsic cell firing frequency increased can be explained by changes in the shape of the sPRC. In networks exhibiting two-cluster firing, the average firing frequency of a cell within this network activity pattern was significantly lower than the average firing frequency of a cell in a network exhibiting either one-cluster firing or full synchrony (Figure 13A). The sPRC for these neurons firing at a frequency observed during two-cluster firing (the 12.4 Hz sPRC in Figure 13B) exhibited slope and skew typical of a sPRC calculated for a Type II neuron. In contrast, the sPRC for these neurons firing at a frequency observed during one-cluster firing (the 55 Hz PRC in Figure 13B) showed a strong phase-resetting shape. Thus, both types of network dynamics exhibited in networks of Type II neurons with adaptation are predicted by variations in the shapes of their sPRCs with increasing intrinsic cell firing frequency. The sPRCs for Type I neurons and Type II neurons, on the other hand, do not show significant variation to their overall shapes and skews in response to changes in the cell's firing frequency that correspond with the range of average network cell firing frequencies exhibited in our simulations (Figures 13C–F), thus predicting the robustness of one-cluster or two-cluster dynamics in these networks, respectively. This frequency-dependence of PRC shape for Type II neurons with adaptation has previously been discussed in relation to the effects of the M-current (Ermentrout et al., 2001; Ermentrout and Wechselberger, 2009; Fink et al., 2011; Ladenbauer et al., 2012), although the specific effects on the dynamics of inhibitory networks of the type studied here have not.

The behavior of both the one-cluster and two-cluster dynamics exhibited by networks of Type II neurons with adaptation differed in important ways from analagous behavior in networks of Type I or Type II neurons, respectively. The adaptation current in the cells of these networks generated unique characteristics of clustered firing. Specifically, networks of Type II neurons with adaptation that displayed one-cluster firing exhibited, on average, clusters containing more neurons than the single clusters in Type I networks in the high heterogeneity case, which is reflected in the higher value of *S* seen in these networks. Additionally, all neurons fired in a majority of the clusters if the network contained Type II neurons with adaptation, while in networks of Type I neurons many cells fired in every cluster while many others were completely suppressed. This difference is illustrated by comparing the average firing frequencies of individual neurons as a function of the input current to those neurons in similar example networks shown in Figures 7A,C.

**Figure 14:**
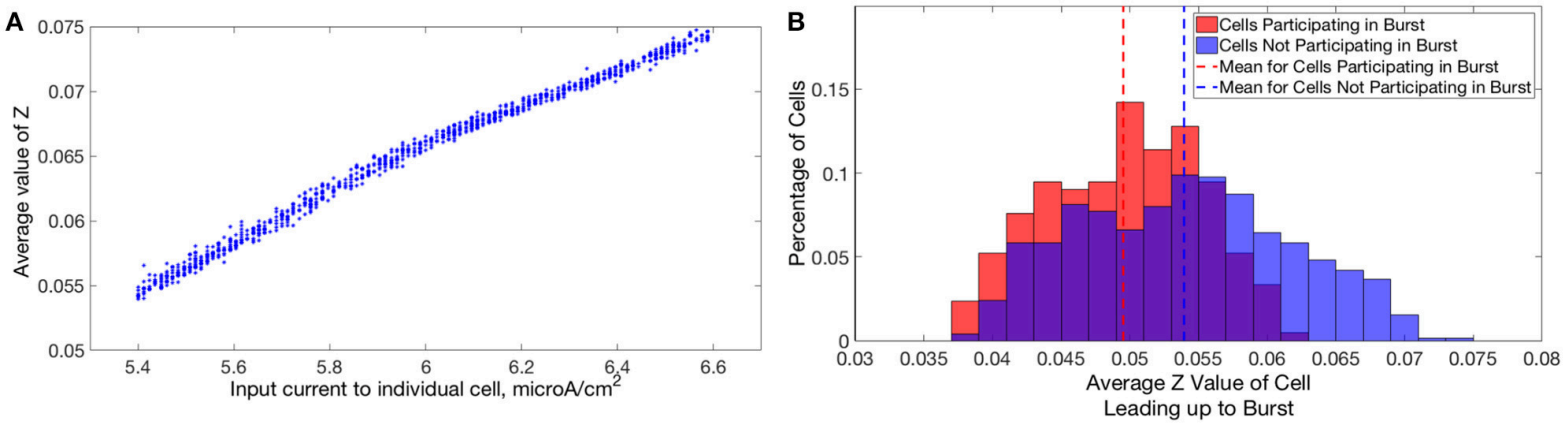
**Dynamics of adaptation current explains cell firing activity in networks of Type II neurons with adaptation exhibiting one or two-cluster dynamics**. **(A)** Average value of the slow potassium gating variable *z* plotted against the input current to each neuron in a network of Type II neurons with adaptation exhibiting one-cluster dynamics. **(B)** Histogram of average *z* values of neurons leading up to a particular burst of activity in a network of Type II neurons with adaptation exhibiting two-cluster dynamics, differentiating neurons participating in the burst (red) and those that are quiescent during that burst (blue).

In Type I networks, since the time interval between cluster firings was primarily determined by the duration of synaptic inhibition, slower firing cells were not able to escape inhibition and fire before the faster firing cells initiated the next cluster firing. As a result, the slower firing cells did not fire in every cluster burst and were often completely suppressed. However, in networks of Type II neurons with adaptation exhibiting one-cluster dynamics, no cells were completely suppressed because deactivation of the slow potassium current makes cells more excitable following extended periods of quiescence. In these networks, cells with lower *I*_*app*_ values did not participate in every cluster firing, so the slow potassium gating variable *z* decayed to lower values between spike firings, as evidenced by their lower average *z* value compared to cells with higher *I*_*app*_ values (Figure 14A). Consequently, at the time of subsequent bursts, these cells were more excitable and were able to escape inhibition and fire with the faster firing cells that initiate cluster firing. Thus, the adaptation current serves to minimize the “effective heterogeneity” of these networks by minimizing the variability in the firing frequencies of individual neurons within the network.

The adaptation current also influenced the pattern of cell firing in two-cluster dynamics. In particular, the clusters in networks of Type II neurons with adaptation were not identical over time, as shown by the fact that neurons in such a network exhibited a range of average firing frequencies dependent upon their input currents, as shown in Figure 7B. In contrast, neurons in networks of Type II neurons that displayed two-cluster dynamics exhibited identical individual neuron firing frequencies irregardless of the neuron's input current (Figure 7D), which indicates that the clusters in these networks were stable.

Furthermore, such networks of Type II neurons with adaptation did not segregate into clusters based upon the neurons' *I*_*app*_, unlike those formed by networks of Type II neurons. Again, effects of the adaptation current on neuron frequency were responsible: frequency of these cells is variable over time, dependent upon the amount of firing that has occurred in the recent past. Cells that participated in a burst had a lower value of the slow-potassium gating variable *z* leading up to the beginning of a burst compared to cells that were quiescent during that burst. Furthermore, cells with the highest *z* values did not participate in the burst. This is illustrated by the histograms in Figure 14B: the red histogram shows the average *z* values of cells participating in a particular burst in the moments before the burst occurred, while the blue histogram shows the average *z* values of cells not participating in the burst. The offset between these histograms implies that whether a cell fires in a given cluster depends strongly on spike-frequency adaptation and not just a cell's external input current. The large overlap between the two histograms may be due to the randomness in total synaptic inhibitory input arriving at each cell given the random connectivity.

In summary, the presence of the adaptation current allowed for effective switching between the dynamics exhibited by Type I and Type II networks. Furthermore, the adaptation current minimized the effective heterogeneity present in one-cluster dynamics while preventing neurons from segregating into unique clusters when exhibiting two-cluster dynamics, distinguishing the dynamics in these networks from similar dynamics in networks of Type I neurons or Type II neurons.

## 4. Discussion

We have shown that intrinsic cellular properties, especially properties of the PRC and the presence of an adaptation current, are of paramount importance in the synchrony and clustering dynamics of a randomly connected network of inhibitory neurons. Furthermore, these intrinsic cellular properties can be the driving force behind potential mechanisms causing these dynamics.

These effects were highly dependent on the degree of connectivity within these networks. Increasing connectivity density limited the contribution of the intrinsic cell firing frequency and synaptic decay constant in determining network dynamics. Additionally, increasing connectivity density changed the type of clustering dynamics the networks exhibited. Most crucially, networks of Type II neurons with adaptation exhibited two-cluster firing for networks with low intrinsic cell firing frequencies and lower connectivity densities, but one-cluster dynamics when the network had higher connectivity density. Furthermore, in networks of Type I neurons, lower connectivity density allowed for bistability between asynchronous and one-cluster dynamics when intrinsic cell firing frequencies were low. When the connectivity density was high, networks evolved directly into one-cluster firing.

In this study, we focused on networks with 30% connectivity density since there is evidence for this level of connectivity among interneurons in the hippocampus (Ascoli and Atkeson, 2005; Viriyopase et al., 2016) and this level displayed dynamics distinct from both very sparsely connected and fully connected networks, as shown in Figure 4. Our focus on this connectivity regime necessitated a numerical study. Previous studies have applied analytical techniques, such as reduction to phase oscillator models, to the investigation of interneuron network dynamics (Vreeswijk et al., 1994; Hansel et al., 1995; Achuthan and Canavier, 2009; Ermentrout and Wechselberger, 2009; Zahid and Skinner, 2009; Ladenbauer et al., 2012); however, these techniques rely on assumptions of all-to-all connectivity and weak coupling among neurons. Our results clearly violate these assumptions: the network regimes we focus on exhibit different dynamics from those observed with 100% connectivity density and the suppression of cell firing observed in the one-cluster dynamics of networks of Type I neurons (see Figure 7B) contradicts the hypotheses of the weak coupling regime.

Furthermore, much of the existing literature analyzing networks of interneurons has focused on gap-junctional coupling as opposed to synaptic inhibition (Ermentrout and Wechselberger, 2009; Zahid and Skinner, 2009). Gap-junctional coupling is instantaneous and can be both excitatory and inhibitory, while synaptic inhibition is purely inhibitory and possesses an intrinsic timescale. These studies also investigate all-to-all coupled networks with weak coupling between neurons, which can be analyzed using techniques such as the phase-reduction method and weakly-coupled oscillator theory (Schemer and Lewis, 2012).

We have illustrated that while networks of Type I neurons exhibit full synchrony or one-cluster dynamics driven by the classical ING mechanism, which relies upon properties of the synaptic current, networks of Type II neurons exhibit two-cluster dynamics driven by neuronal excitability properties (namely, the concave down shape of these neurons' sPRCs). Additionally, networks of Type II neurons with adaptation displayed either one-cluster or two-cluster dynamics dependent upon the average firing frequency of neurons in the network and the effect this frequency had on the properties of the sPRCs of these cells.

While low heterogeneity networks of Type II neurons fully synchronized via the ING mechanism, these networks exhibited two-cluster dynamics for short lasting inhibitory synapses in low heterogeneity networks and for nearly all values of the synaptic decay time constant with high heterogeneity. Neurons forming these clusters were segregated based upon their natural firing frequencies. The network stabilized two-cluster dynamics by forming asymmetric timing of cluster firings that, due to the skew of the sPRC, led to a different magnitude of delay experienced by each respective cluster. This asymmetry acted to balance the differences in natural firing frequencies of neurons in each cluster.

The tendency for inhibitory networks containing Type II neurons to display the two-cluster dynamics observed here has been previously seen in studies looking at small networks, all-to-all connected networks, and networks containing other methods of signal propagation than synaptic inhibition (Vreeswijk et al., 1994; Hansel et al., 1995; Achuthan and Canavier, 2009; Ladenbauer et al., 2012; Viriyopase et al., 2016). However, given the importance of the degree of network connectivity in determining clustering dynamics shown in this study, it can not merely be assumed that these dynamics extend to a larger, randomly coupled network. Our simulations justify this extension. Furthermore, our analysis revealed that heterogeneity in intrinsic firing frequencies can be compensated for by differences in firing times of each cluster and of individual neurons within in each cluster in order to promote two-cluster dynamics in these networks.

Networks of Type II neurons with adaptation exhibited behavior similar to the one-cluster dynamics of networks of Type I neurons or the two-cluster dynamics of networks of Type II neurons, dependent upon how the average intrinsic cell firing frequency and synaptic decay constant dictated the average firing frequency of cells in the network. When neurons in the network fired sufficiently fast, the network behaved similarly to networks of Type I neurons, because the sPRC of Type II neurons with adaptation computed at such frequencies mirrored the phase-resetting properties of a Type I neuron. However, the one cluster formed in such a network of Type II neurons with adaptation contained more active neurons, on average, then similar clusters formed in networks of Type I neurons. This difference was caused by the influence of the adaptation current in increasing the excitability of neurons following a period of quiescence.

When cells in a network of Type II neurons with adaptation fired more slowly, the network exhibited behavior similar to the two-cluster dynamics shown by networks of Type II neurons, because the sPRCs calculated for Type II neurons with adaptation firing at this slower frequency matched the shape and skew properties, in particular the concave down nature, of sPRCs of Type II neurons. Here the adaptation current also played a pivotal role in differentiating the dynamics in networks of Type II neurons with adaptation from those of networks of Type II neurons. In particular, the changing cellular excitability of Type II neurons with adaptation brought about by the adaptation current prevented the segregation of neurons into unique clusters based upon their *I*_*app*_ values, as was the case in networks of Type II neurons.

We note that care should be taken when interpreting PRCs for neuron models that contain active currents with slowly evolving gating variables, like the M-current in our Type II neuron with adaptation model. Specifically, perturbations can have effects on firing cycles subsequent to the cycle in which the perturbation occurred. To account for such longer-lasting effects of the perturbation, previous studies have employed higher-order PRCs (Oprisan et al., 2004; Talathi et al., 2009) and functional PRCs (Cui et al., 2009). Indeed, for our Type II neuron with adaptation, the perturbation used to compute the sPRC did result in slightly shorter periods for several firing cycles subsequent to the perturbation cycle due to the influence of spike-frequency adaptation. For our Type I and Type II model neurons, on the other hand, firing cycles subsequent to the perturbation cycle showed no effects of the perturbation.

Thus, for our networks of Type II neurons with adaptation we have focused on applying the sPRC to explain the transition from one-cluster dynamics to two-cluster dynamics by considering the change in its overall shape as the firing frequency increases: from a more concave down shape to the more linear phase resetting shape as firing frequency increases. As shown in Figures 13A,B, the correspondence of the change in sPRC shape with the change in network frequency and thus cluster dynamics is remarkably tight: the low network frequency parameter regimes (blue in Figure 13A) correspond to concave down sPRCs (blue curves in Figure 13B) and display two-cluster dynamics, while the high network frequency parameter regimes (green and warmer colors in Figure 13A) correspond to phase resetting sPRCs (green and warmer color curves in Figure 13B) and exhibit one-cluster dynamics. We then highlight features of the time dynamics of the M-current gating variable to explain how cell participation in the one-cluster and two-cluster dynamics differs from the Type I and Type II networks, respectively, in Figures 7, 14. For our networks of Type II neurons, since the sPRC is the same for all cycles, we are able to expand its use to understand the segregation of neurons between the two-clusters and to explain the asymmetric timing pattern of the firing of the two-clusters, as shown in Figure 10.

Our results for networks of Type I neurons paralleled those of previous works in the field, including the work of Wang and Buzsáki (1996) and Whittington et al. (2000), as well as more recent, biologically driven simulations of Type I neurons by Ferguson et al. (2013). In simulating similar networks of Type I neurons with heterogeneity, Wang and Buzsaki identified a length of synaptic decay that leads to optimal network synchronization. In similar simulations with a model of the PV interneuron (which exhibits distinctly Type I properties), Ferguson et al. showed that synchrony of these networks improves with faster firing neurons. In our results, the application of the synchronizing current pulse revealed that synchrony and one-cluster dynamics are possible in these networks when intrinsic cell firing frequencies are low, but the network may not evolve to those dynamics from random initial conditions. In particular, if only network dynamics as evolved from random initial conditions are considered, it would appear that there is a strict threshold in intrinsic firing frequency for synchronous or one-cluster firing to occur (left panels of Figures 6A,B, 8A,B). The effect of the synchronizing current pulse mimics the conditions for synchronization by the ING mechanism. Specifically, as Whittington states, ING synchrony will occur if enough neural firing occurs in close temporal proximity in order to send a sufficiently strong inhibitory signal throughout the network, which prevents any neuron from firing until this synaptic signal decays (Whittington et al., 2000). While an instance of enough neurons firing in close temporal proximity is likely to happen when intrinsic neuron firing frequencies are high, it is less likely in networks of slower firing cells. For such networks a single, brief stimulation can be enough to induce stable synchronous dynamics.

A brief synchronizing stimulus may provide a mechanism, both experimentally and computationally, by which the presence of ING can be directly assessed. Indeed, the ING theory predicts that if ING-driven dynamics are at all possible for a given network, the instance of synchronous firing caused by the current pulse should always induce ING-driven clustering or synchrony. The fact that the current pulse had minimal effect on networks exhibiting two-cluster dynamics, and never induced two-cluster dynamics from a previously asynchronous network, thus suggests that the ING mechanism does not drive two-cluster dynamics in the networks studied here.

Bistability between asynchronous and synchronous solutions in small networks of two mutually coupled inhibitory neurons, where the initial conditions of the network determine the dynamics of the system, has been previously reported (Terman et al., 1997). Our results serve as a generalization of this phenomena to a larger network with a more complicated connectivity structure.

Our results for networks of Type II neurons are similar to those found for pairs of Type II neurons coupled by mutual inhibition studied by Vreeswijk et al. (1994). They analytically showed that for sufficiently long lasting synapses, anti-synchrony is the stable state of these neurons. Anti-synchrony of two neurons corresponds with the two-cluster dynamics seen in our simulations. The work of Achuthan and Canavier on all-to-all coupled inhibitory networks with four Type II neurons also showed the tendency of these networks to exhibit two-cluster dynamics predicted by properties of the PRC (Achuthan and Canavier, 2009). The tendency for larger networks to exhibit these properties has been shown in work by Ladenbauer et al. and Viriyopase et al., albeit in networks with different connectivity and heterogeneities than those studied here (Ladenbauer et al., 2012; Viriyopase et al., 2016). Our work shows that the results found in these studies can be extended to randomly connected inhibitory networks with heterogeneity in the external input currents to the neurons in the network. We additionally explain intricacies of the two-cluster dynamics, such as the segregation of neurons into unique clusters based upon their intrinsic firing frequency and the asymmetric timing of the cluster firing.

Our results indicate that the clustering properties of Type II neurons, when subjected to high heterogeneity in their external input currents, do not show significant change in response to a change in average intrinsic cell firing frequency or synaptic decay constant. Given that biologically plausible inhibitory networks typically receive a heterogeneous driving current based upon the drive from a network of excitatory neurons, these results imply that the two-clustering properties of a network of Type II interneurons might prove especially robust to changes in the excitatory drive from the network. We found that networks of Type II neurons synchronize fully only when cellular heterogeneity was low and for sufficiently long lasting synapses. This result contradicts previous research that suggested that neurons with Type II properties could not exhibit synchronous behavior in an inhibitory network (Hansel et al., 1995; Achuthan and Canavier, 2009). Recently, work by Tikidji-Hamburyan et al. analyzed a randomly connected network of Type II neurons that exhibit post-inhibitory rebound firing (which neither our Type II or Type II with adaptation models exhibit). Their results illustrated that such networks can form synchronous gamma rhythms in a fashion more robust to heterogeneity than similar networks of Type I neurons, driven primarily by the properties of the post-inhibitory rebound firing (Tikidji-Hamburyan et al., 2015).

The results for networks of Type II neurons with adaptation are of particular biological relevance, considering that the OLM interneurons of the hippocampus exhibit the M-type adaptation current (Saraga et al., 2003; Lawrence et al., 2006; Cutsuridis et al., 2010; Cutsuridis and Hasselmo, 2012), as do some interneurons in the cortex (Markram et al., 2004; Perrenoud et al., 2013). We have shown that the presence of the adaptation current imbues these neurons with clustering properties and a mechanism driving these dynamics that is distinct from that of Type II neurons without adaptation.

The concentration of ACh in the brain is strongly correlated with sleep state, with the concentrations at their highest during wake and REM sleep. Additionally, the important role of ACh in the hippocampus and its effect on M-type potassium channels has been well studied (Aton et al., 2013; Teles-Grilo Ruivo and Mellor, 2013). Our results provide a potential mechanistic explanation for how ACh can affect pattern generation amongst networks of interneurons; in particular, we have shown that inhibitory networks comprised of neurons containing an M-current will exhibit only one-cluster dynamics when the ACh concentration is high, blocking the M-current and making the neuron Type I, while these networks may exhibit two-cluster dynamics when the ACh concentration is low and the M-current is active. Additionally, the ability for these networks to exhibit either two-cluster or one-cluster dynamics, largely dependent upon how much applied current drives the network, could provide a “gate” by which inhibitory tone to downstream neurons is modulated. These effects on pattern generation might potentially be propagated to pyramidal cells and affect the overall oscillatory behavior of the hippocampus and cortex.

## Author contributions

Simulations performed by SR. Analysis of results and articulations of underlying mechanisms performed by SR, VB, and MZ. Paper written and edited by SR, VB, and MZ.

## Funding

Research funded in part by grants: NIH NIBIB 1R01EB018297 (SR, VB, and MZ) and NSF PoLS 1058034 (MZ).

### Conflict of interest statement

The authors declare that the research was conducted in the absence of any commercial or financial relationships that could be construed as a potential conflict of interest. The reviewer LS and handling Editor declared their shared affiliation, and the handling Editor states that the process nevertheless met the standards of a fair and objective review.
